# Optimized Synthesis of Solution‐Processable Crystalline Poly(Triazine Imide) with Minimized Defects for OLED Application

**DOI:** 10.1002/anie.202111749

**Published:** 2021-12-18

**Authors:** David Burmeister, Ha Anh Tran, Johannes Müller, Michele Guerrini, Caterina Cocchi, Julian Plaickner, Zdravko Kochovski, Emil J. W. List‐Kratochvil, Michael J. Bojdys

**Affiliations:** ^1^ Department of Chemistry Kings College London Britannia House Guy's Campus, 7 Trinity Street London SE1 1DB United Kingdom; ^2^ Department of Chemistry Department of Physics IRIS Adlershof Humboldt-Universität zu Berlin Zum Grossen Windkanal 2 12489 Berlin Germany; ^3^ Department of Physics IRIS Adlershof Humboldt-Universität zu Berlin Brook-Taylor-Strasse 15 12489 Berlin Germany; ^4^ Institute of Physics Carl von Ossietzky Universität Oldenburg 26129 Oldenburg Germany; ^5^ Helmholtz-Zentrum Berlin für Materialien und Energie GmbH Hahn-Meitner-Platz 1 14109 Berlin Germany; ^6^ Leibniz-Institut für Analytische Wissenschaften—IAS e.V. Schwarzschildstrasse 8 12489 Berlin Germany; ^7^ Institute of Electrochemical Energy Storage Helmholtz-Zentrum Berlin für Materialien und Energie Hahn-Meitner-Platz 1 14109 Berlin Germany

**Keywords:** covalent organic framework, crystalline carbon nitride, ionothermal synthesis, layered materials, metal-free semiconductor

## Abstract

Poly(triazine imide) (PTI) is a highly crystalline semiconductor, and though no techniques exist that enable synthesis of macroscopic monolayers of PTI, it is possible to study it in thin layer device applications that are compatible with its polycrystalline, nanoscale morphology. We find that the by‐product of conventional PTI synthesis is a C−C carbon‐rich phase that is detrimental for charge transport and photoluminescence. An optimized synthetic protocol yields a PTI material with an increased quantum yield, enabled photocurrent and electroluminescence. We report that protonation of the PTI structure happens preferentially at the pyridinic N atoms of the triazine rings, is accompanied by exfoliation of PTI layers, and contributes to increases in quantum yield and exciton lifetimes. This study describes structure–property relationships in PTI that link the nature of defects, their formation, and how to avoid them with the optical and electronic performance of PTI. On the basis of our findings, we create an OLED prototype with PTI as the active, metal‐free material.

## Graphical abstract

The effects of low‐energy defects, charge delocalization, and exfoliation on the optical and electronic performance of a layered, metal‐free intercalation compound, poly(triazine imide) (PTI) are reported. Using an adjusted synthetic protocol, the quantum yield and the photocurrent of the semiconducting PTI could be increased. Due to the high quality of the product, structure–property relationships could be studied and PTI could be integrated in a single‐layer OLED.

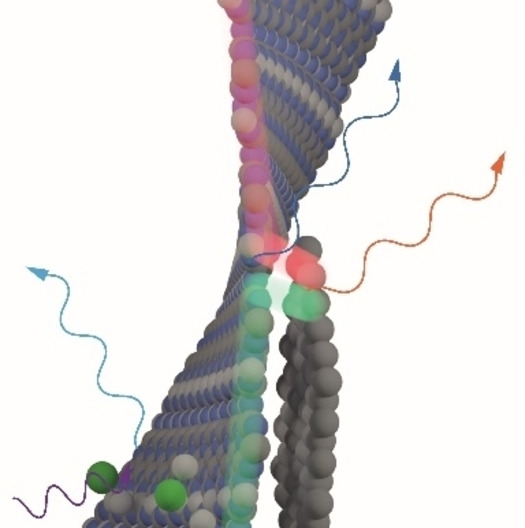

## Introduction

Interest in atomically thin 2D materials skyrocketed with the isolation of graphene sheets from graphite parent crystals and the realization that these sheets can be exploited in optoelectronic devices.^[1]^ The extraordinarily high charge carrier mobility in graphene of up to 200 000 cm^2^ V^−1^ s^−1^ is a result of the changed electronic structure of the atomic layer when compared to the bulk material.^[2]^ The high mobility of semimetallic graphene comes at the price of an absent “off state” that has foreclosed its application in transistors.[^3^, ^4^] Strategies to open up graphene's band gap revolve around the introduction of heteroatoms and structural defects in diffusion‐limited or statistical post‐processing as well as nanostructuring.[^5^, ^6^, ^7^] However, none of these methods are scalable and they do not yield products able to compete with silicon‐based devices to date.^[8]^

Crystalline organic 2D materials that contain predictable “deletions” (i.e. pores) and heteroatoms by design are researched to make up for the missing band gap of graphene. Most notably transition metal dichalcogenides, graphene derivatives and designer covalent organic frameworks (COFs) compete to be the next generation semiconductor.[^8^, ^9^, ^10^, ^11^, ^12^]

Graphitic carbon nitrides combine two important characteristics: (i) they are overall sp^2^‐hybridized and covalently bonded, and (ii) they consist of earth abundant, light elements (i.e. they are metal free).[^13^, ^14^] Poly(triazine imide) (PTI‐MX) is a layered intercalation compound obtained from alkali metal halide salt melts and can be exfoliated to monolayers.^[15]^ Even though the material has high environmental stability, crystallinity, blue photoluminescence, and has found application as semiconductor in photo‐ and electrocatalysts, it has found little to no use in optoelectronic devices up to this point.[^16^, ^17^, ^18^] Herein, (i) we elucidate the impact of different polycondensation conditions on the chemical makeup of PTI and its optical and electronic performance, (ii) we monitor the material quality at microscopic and macroscopic scales, (iii) we assemble photoconductor and organic light emitting diode (OLED) devices based on PTI, and (iv) we look at the effect of protonation on the electronic structure of PTI comparing results from calculations with observed optical spectra. The results of this study provide guidelines for improving the optical and electronic properties of organic layered materials; especially ones that incorporate triazine (C_3_N_3_) subunits.

Ionothermal condensation–polymerization of dicyandiamide was first performed by Bojdys et al., and later, Wirnhier et al. solved the structure of the product as poly(triazine imide).[^19^, ^20^] While the structure of this material was examined in detail, the electronic properties of this system have not been studied thoroughly yet.[^19^, ^21^, ^22^, ^23^] We find that the reduction of the temperature increases the structural order and preserves a desired CN‐ratio. This enables us to harness the semiconducting properties in simple prototype devices. To access quality markers and properties of the obtained product we applied powder X‐ray diffraction (PXRD), scanning electron microscopy (SEM), X‐ray photoelectron spectroscopy (XPS), ultraviolet photoelectron spectroscopy (UPS), Fourier‐transform infrared spectroscopy (FT‐IR), UV–Raman, solid state nuclear magnetic resonance spectroscopy (ssNMR), UV/Vis absorption spectroscopy (UV/Vis), photoluminescence, and photoluminescence excitation spectroscopy (PL, PLE) as well as quantum yield and lifetime measurements.

The observations of this work also are of explanatory value (i) for the wider organic materials community studying the effects of defects in conjugated polymer semiconductors and (ii) in the context of wide‐spread studies into the photocatalytic activity of PTI.^[24]^

## Results and Discussion

### Structural Characterization of Obtained Products

**Figure 1:**
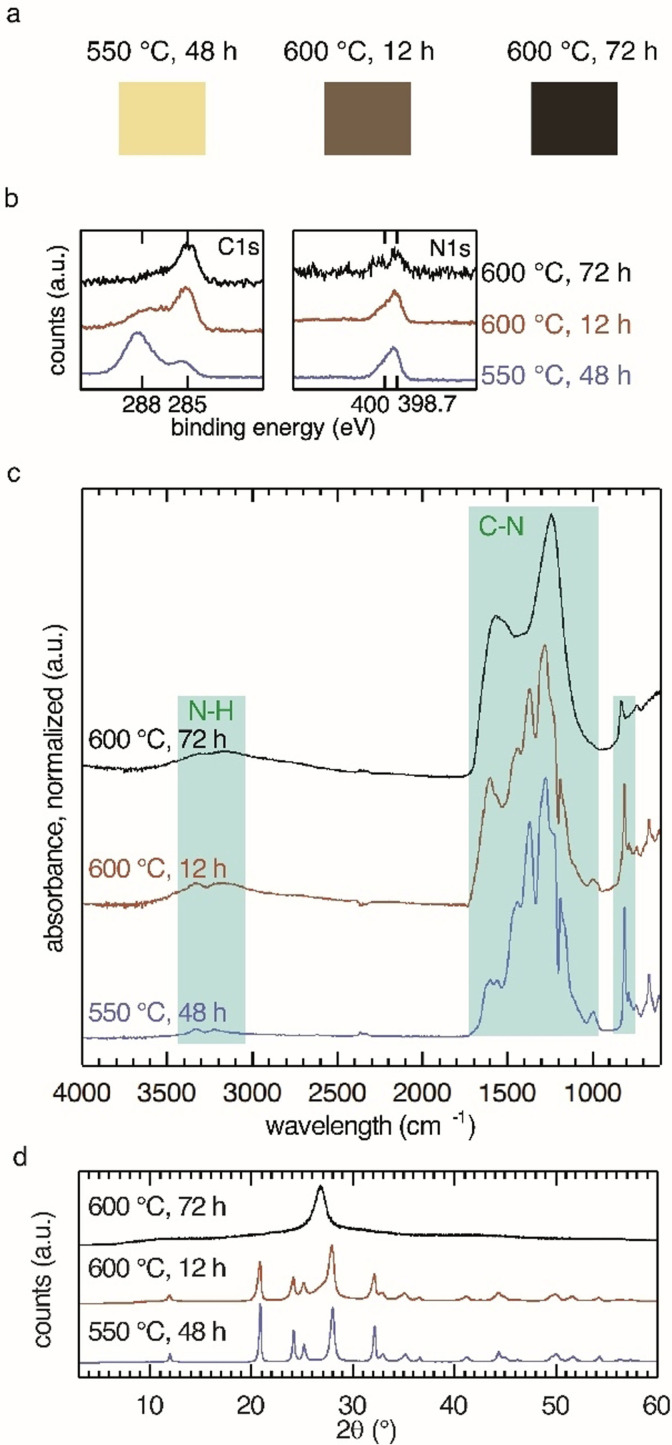
Structural characterization of PTI–LiBr materials obtained at different temperatures and reaction times. a) Discoloration of PTI–LiBr with increasing reaction temperature indicates emergence of carbon‐rich phase for products formed at 600 °C. b) X‐ray photoelectron spectroscopy (XPS) of PTI–LiBr samples. Evaluation of C1s and N1s core regions confirms the presence of carbon‐rich product and loss of nitrogen at 600 °C. c) Fourier‐transform infrared (FT‐IR) spectra of PTI–LiBr products show decreased band separation for high reaction temperatures, indicating the loss of structural order. d) Powder X‐ray diffraction pattern of product phases. The evolution of a peak at 27° 2*θ* for products formed at 600 °C is interpreted as formation of a disordered carbon‐rich material next to PTI–LiBr.

The starting point for this work is the ionothermal synthetic protocol for PTI that has not been altered since its first reports.[^25^, ^26^, ^27^] The significant parameters for polycondensation reactions are the reaction temperature (600 °C) and the reaction time (12 h). The product is characterized as a dark brown powder (Figure 1 a, center).^[28]^ The material shows no visible photoluminescence under 375 nm UV‐light (Figure S1). Higher luminescence can typically be accessed only by dispersing the material in water/methanol/DMSO.^[27]^ Hence, it is often argued that the emission of the material in its initial graphitic state is quenched by π–π stacking. Instead of excitation and radiative emission, π–π quenching leads to photo‐induced electron transfer to a neighboring π‐system. This inhibits effective recombination of the electron hole pair in a radiative process.

We report that synthesis at a temperature of 550 °C and extended reaction times of 48 h yields an almost colorless product (Figure 1 a, left) with visible photoluminescence when irradiated with UV‐light. The hitherto employed harsh reaction conditions (600 °C, 12 h) are at the onset of carbonization of the material and introduce carbonaceous contaminants that darken the color of PTI.^[29]^ Qualitative and quantitative XPS analysis of the C1s core region shows an intense peak at 285 eV and confirms the presence of C−C environments (adventitious C1s, C−C peak at 284.8 eV) (Figure 1 b).^[30]^ This contribution is often assigned to adventitious carbon.[^25^, ^31^, ^32^]

However, at 550 °C the contribution at 285 eV in the C1s spectrum of PTI–LiBr is greatly reduced. Since the contribution of adventitious carbon should be similar for all samples, we deduce that part of this contribution comes from a carbon‐rich phase that coevolved during the synthesis at 600 °C. Looking at the N1s region, we observe nitrogen signals from triazine moieties at 398.5 eV and a contribution from the imide‐NH bridges between the triazine units at 400 eV with relative intensities of (3:1) for the product from 550 °C (Figure 1 b, right). The product obtained at 550 °C, 48 h approaches the theoretical elemental composition of poly(triazine imide) (C_6_N_9_H_3_) of 40:60 C:N most closely (Table 1). Other groups have not reported the presence of this carbon‐rich phase at these conditions. Hence it is likely that it was either overlooked or mistaken for a contribution solely from carbon tape used as substrate or adventitious carbon in XPS experiments. An attempt to study the structure of the phase obtained from 600 °C for 72 h was conducted by Suter et al.^[33]^

**Table 1:** X‐ray photoelectron spectroscopy (XPS) analysis of atomic percentage of carbon and nitrogen observed in CN films prepared from different synthetic conditions.^[a]^

	Reaction conditions	C:N at % XPS	
	600 °C, 72 h	87:13	
	600 °C, 12 h	81:19	
	550 °C, 48 h	49:51

[a] The integration of the carbon signal was conducted over all carbon species and not corrected for adventitious carbon.

To access information about the prevalent bond types and therefore the degree of condensation as well as possible defects, we conducted FT‐IR spectroscopy on the products (Figure 1 c). FT‐IR spectra of the high‐temperature products at 600 °C show a broad peak around 3300 cm^−1^ in addition to two discernible NH signals (at 3328 and 3217 cm^−1^). The latter are argued to originate from splitting of the imide N−H bond by partial conversion into N−Li.^[19]^ The broad feature is absent in the product obtained at 550 °C. The sharp band around 810 cm^−1^ is attributed to the out‐of‐plane bending mode of the six‐membered triazine (C_3_N_3_) ring and is present in all samples.[^34^, ^35^, ^36^] Likewise, the material obtained at low temperature has a clearer separation of bands in the CN stretching region 990–1600 cm^−1^. There are two valid explanations for these observations: (i) the broad feature around 3300 cm^−1^ can be attributed to OH groups attached to the carbonaceous contaminants that are produced at elevated temperatures, and (ii) decreased band separation is indicative of structural disorder in the material obtained at high temperature as a consequence of thermal decomposition. The spectra of samples condensed at 600 °C for 72 h resemble those of amorphous CNH‐compounds with the dominant infrared absorption between 1600 and 1300 cm^−1^ from C−N modes and ring species.^[37]^

PXRD patterns confirm the transition of crystalline PTI–LiBr into a disordered material at long reaction times and 600 °C (Figure 1 d). Condensation at 550 °C results in a slight shift in the diffraction pattern towards lower angles corresponding to an increase in plane distances. A conceivable reason is an increased ion loading in the structure obtained at reduced temperature.^[38]^ The same trend of higher product quality at the lower reaction temperature was also found by ssNMR. The C^13^ spectrum shows the three carbon signals also observed in PTI–LiCl at *δ*=167.0 ppm, 162.0 ppm and 158.0 ppm (Figure S2). The bands show increased sharpness in the product condensed at 550 °C.^[19]^

We examined the sample morphologies by SEM (Figure S3 a,b). The morphology of PTI‐MX is described as hexagonal platelets and prisms with a basal cross‐section of 100–500 nm in literature.[^20^, ^39^] The nanostructure has been elucidated by high resolution transmission electron microscopy (HR‐TEM, Figure S3 c–e). Fourier transformation of the HR‐TEM image reveals the hexagonal periodicity and the characteristic in‐plane distance of 0.73 nm corresponding to the 100 reflection at 12° 2*θ* in the PXRD spectrum (Figure 1 d) as indexed by Suter et al.^[25]^

Further decrease of the reaction temperature to 525 °C resulted in a product with additional PXRD peaks that could not be attributed to a PTI‐type material and, hence, the material was not investigated further (Figure S4). We selected the PTI–LiBr sample obtained at 550 °C (48 h) for further optoelectronic experiments based on its structure and composition.

### Optical Properties of PTI–LiBr and PTI‐IF Films

**Figure 2:**
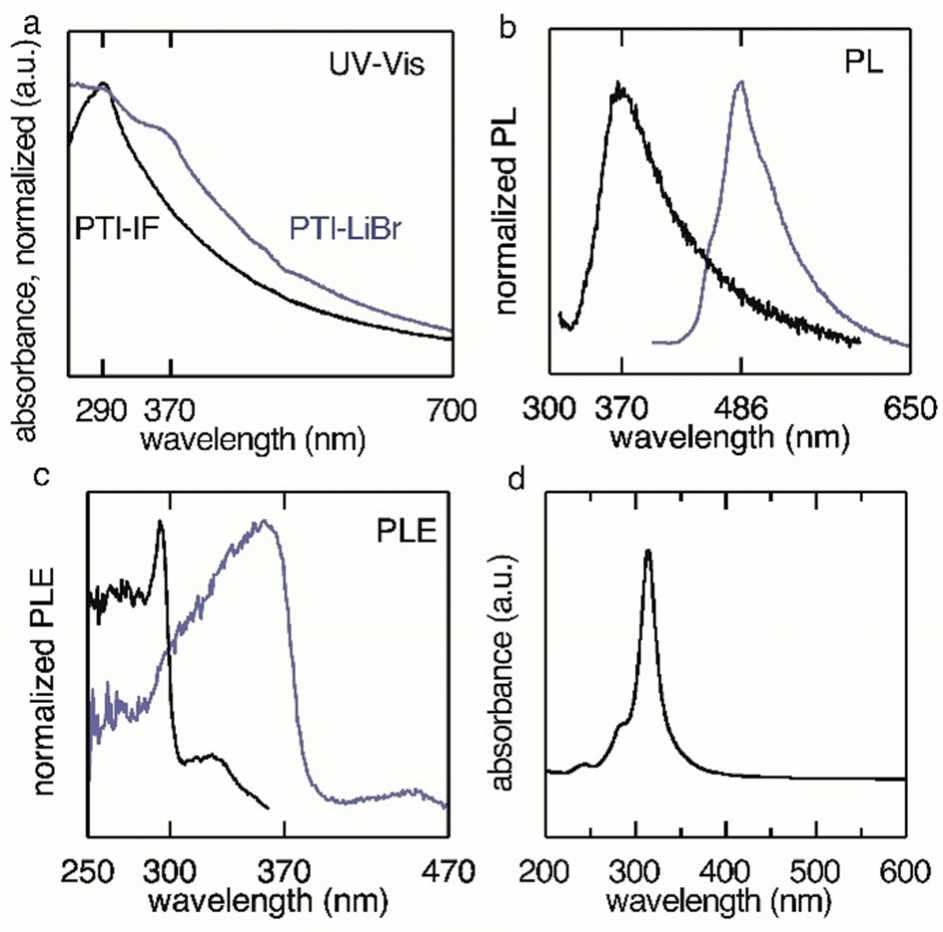
Optical characterization of PTI–LiBr obtained at 550 °C, 12 h (in blue) and intercalation‐free poly(triazine imide) (PTI‐IF, in black) on quartz substrates. a) UV/Vis (transmission) spectrum. b) Photoluminescence spectra show emission maxima at 486 nm for PTI–LiBr and at 370 nm for PTI‐IF. c) Photoluminescence excitation spectra of PTI–LiBr (excited at 370 nm) and PTI‐IF (excited at 300 nm). d) Absorption spectrum of a monolayer of PTI‐IF computed from time‐dependent DFT.

**Figure 3:**
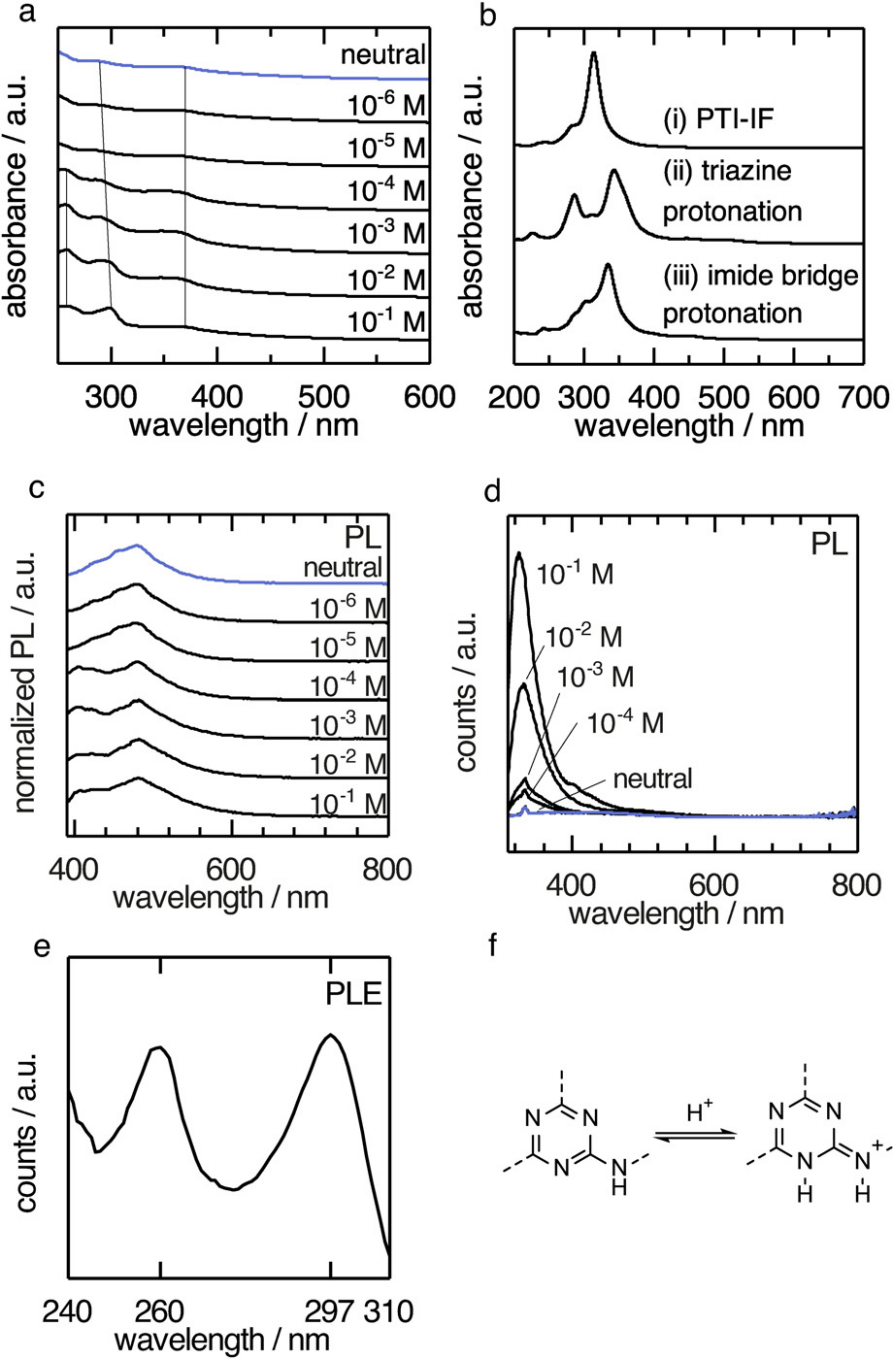
Optical characterization of PTI–LiBr dispersions in water at different concentrations of HCl. a) UV/Vis (transmission) spectra. b) Time‐dependent DFT absorption spectrum of (i) a monolayer PTI‐IF, (ii) PTI‐IF monolayer protonated via HCl at triazine rings and (iii) PTI‐IF monolayer protonated via HCl at imide bridges. c) Photoluminescence (PL) spectra excited at 370 nm for PTI–LiBr dispersions with different HCl concentrations. d) Photoluminescence spectra excited at 300 nm for PTI–LiBr dispersions with different HCl concentrations. e) Photoluminescence excitation (PLE) spectrum for emission at 330 nm at 10^−2^ M HCl. f) Energetically favored protonation at the triazine ring.

The decreased carbon contamination allows us to measure UV/Vis of PTI–LiBr dispersions (Figure 2 a, blue plot). We find two broad features around 370 and 290 nm. The state at 370 nm can be excited yielding the photoluminescence spectrum shown in Figure 2 b (blue plot). The photoluminescence excitation spectrum shows a single maximum at 486 nm (Figure 2 c, blue line). This emissive state is a result of the replacement of hydrogen with lithium at triazine bridging N−H units. This defect reduces the conduction band minimum energy accounting for the contraction of the optical gap of this structure.^[28]^ Exciting PTI–LiBr at around 290 nm does not yield a new emission band and decreases the emission at 486 nm drastically (cf. Figure 3 d). Hence, the state that belongs to the absorption at 290 nm in PTI–LiBr is quenched.

To gain a better understanding of these two states, we prepared an intercalation‐free PTI (PTI‐IF) comprised only of the CN‐backbone (SI Experimental). PXRD, FT‐IR and UV–Raman of PTI‐IF are depicted in Figure S5. Removal of the coordinating lithium and bromine ions leads to disorder as can be found comparing the PXRD spectra of PTI‐IF to PTI–LiBr. The width of the reflexes is broadened and some peaks are deleted entirely. Comparing FT‐IR spectra of PTI–LiBr and PTI‐IF we find better band separation in the ion‐free PTI‐IF as was observed by Suter et al.^[25]^ UV–Raman spectra with a 266 nm excitation source reveal the same trend of sharper bands in PTI‐IF. The Raman bands associated with breathing modes of triazine are found at 1002 cm^−1^ (weak) and 685 cm^−1^ (weak) in PTI–LiBr and 983 cm^−1^ (strong) and 685 cm^−1^ (weak) in PTI‐IF.[^33^, ^40^] Broad contributions in both samples at 1300 cm^−1^ (broad, weak) and 1630 cm^−1^ (strong) can be assigned to the so‐called D and G bands due to breathing modes in sp^2^‐bonded rings and CN sp^2^ bond stretching in rings and chains, respectively.^[37]^ The presence of a sharp, strong Raman band at 1088 cm^−1^ in PTI–LiBr samples indicates a carbonate species.^[41]^ The lithium defect can undergo hydrolysis to form LiOH in presence of water. This is the known reason for the basic character of PTI‐MX in water as reported in literature.^[42]^ LiOH is a known CO_2_ absorber.^[43]^ We propose that under environmental conditions (in presence of water and CO_2_) LiOH can form at the surface of the crystallites which in turn can absorb CO_2_ leading to the presence of lithium carbonate species. Hence, in PTI‐IF this Raman band is absent.

Optical characterization by UV/Vis spectroscopy reveals that by removing most of the ions in the structure, we lose the absorption band at 370 nm, as expected, since this state is the result of Li‐defects. Absorption at 290 nm is still prevalent and, hence, it is assigned to the PTI‐conjugated backbone. This observation matches well with the theoretical predictions for an intercalation‐free PTI (see Figure 2 d). The result of this calculation shows that PTI‐IF layers have a single bright absorption band at around 320 nm, in good agreement with the experimental results, considering the necessary simplifications applied to the model structure adopted in the simulation (see Figure S6, S7) The absence of emission from the 290 nm state in PTI–LiBr could be due to the presence of the Li defects acting as a deep trap. Excitons formed in the organic backbone will recombine primarily at these energetically favorable defect sites. A similar effect can be observed for keto‐defects in polyfluorene backbones.^[44]^

It is not possible to determine the quantum yield of PTI samples obtained at 600 °C because of strong absorption from the accompanying carbon‐rich phase. The quantum yield of films cast from the 550 °C product equals 0.4 %. Optical images comparing products obtained at 550 °C and 600 °C illuminated with a 375 nm LED can be accessed in the SI (Figure S1).

### Effect of Protonation on the Optical States of PTI–LiBr

In a first set of experiments, we collected UV/Vis spectra of PTI–LiBr suspended in water at different concentrations of HCl (Figure 3 a). Without HCl we observe the absorption of the lithium defect states at around 370 nm and the broad absorption of the conjugated PTI‐backbone at around 290 nm. By increasing the H_3_O^+^ concentration, two effects can be identified. First the absorption band of the organic backbone experiences a slight bathochromic shift from 290 to 300 nm. Moreover, a new absorption band at 260 nm arises. There are two possible sites of protonation in the PTI–LiBr structure: at the triazine nitrogen (Figure 3 b), or at the imide nitrogen (Figure 3 b). Protonation at the triazine nitrogen compares best with the observed UV/Vis spectra, and accounts for the bathochromic shift and the manifestation of a new band at 260 nm (compare Figure 3 b, i and Figure 3 b, ii). Our first‐principles calculations predict that the triazine‐protonated structure is more stable than the imide‐bridge‐protonated structure. Based on this finding, we assume that the triazine protonation is prevalent. The calculated optical absorption spectra of the protonated materials (Figure 3 b) indicate an overall red‐shift of the absorption peaks in comparison with PTI‐IF. This result is qualitatively in agreement with earlier predictions on analogous model systems.^[45]^ Specifically the red shift of the main peak in combination with the emergence of a second absorption band in the absorption spectrum of triazine‐protonated PTI (Figure 3 b, ii) is considered an effect of the symmetry breaking of the backbone structure upon protonation. The presence of two absorption bands in the system is in good agreement with two corresponding PLE maxima at 260 nm and 297 nm of the emission at 330 nm (Figure 3 d,e) present in dispersions with HCl concentrations larger than 10^−5^ M at 260 nm and 300 nm (Figure 3 d,e).

It is noteworthy that for excitation at 300 nm in dispersions at 10^−2^ M HCl the quantum yield obtained was 10 % and for excitation at 370 nm 9 %. The emission of the neutral dispersion at 486 nm is comparatively low with a quantum yield of 1 % and no emission for excitation at 300 nm. The increased quantum yield is accompanied by an increase in lifetime (neutral *τ*_1_=0.5 ns ±0.03 ns, *τ*_2_=3.6 ns ±0.13 ns; pH 1 *τ*_1_=1 ns ±0.05 ns, *τ*_2_=4 ns ±0.10 ns). As typical for CN materials a two exponential fit was applied.^[32]^ The increase in quantum yield and lifetime is attributed to partial exfoliation of the layered PTI structure and, hence, reduced π–π stacking. Charging of PTI layers by reduction with sodium naphthalide and stabilization by derivatization with a C_12_ alkane was described in literature.^[46]^ A route via protonation could be a viable alternative.

The photoluminescence band we assign to the CN backbone protonated at the triazine moiety at 330 nm is blue‐shifted with respect to the emission of PTI‐IF at 370 nm (Figure 3 d, 2 b).

**Figure 4:**
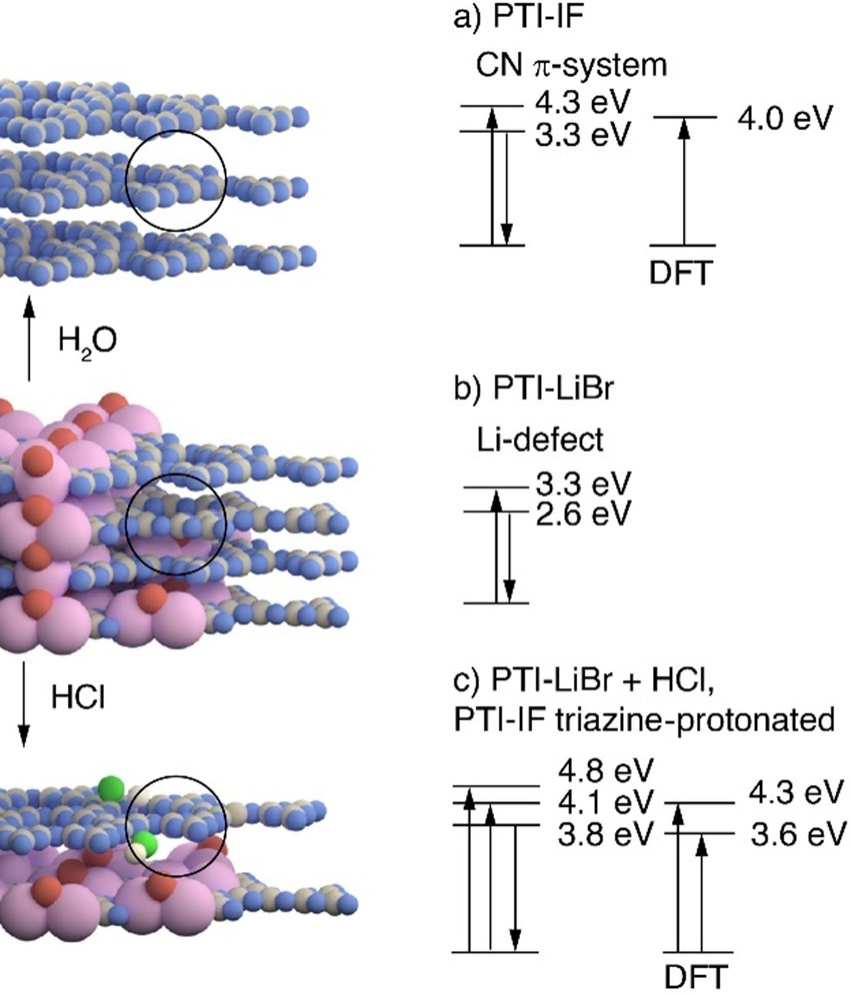
Summary of optical states from DFT, UV/Vis and photoluminescence spectroscopy of dispersions in water of (a) PTI‐IF obtained from PTI–LiBr by Soxhlet extraction of ions, and optical gap of 2D layer of PTI‐IF from DFT calculation, (b) partially de‐intercalated PTI–LiBr as obtained from ionothermal synthesis, (c) effects of HCl at concentrations higher than 10^−5^ M on PTI–LiBr dispersions and optical gaps obtained for 2D layer of triazine‐protonated PTI‐IF sheet by DFT. Arrows pointing upwards denote an absorption process, arrows pointing downwards denote an emission process. Carbon and nitrogen atoms are represented as gray and blue spheres. Hydrogen atoms at the imide nitrogen are not depicted. Lithium and Bromine ions are represented as red and pink spheres. Proton and chloride anion are represented as white and green spheres.

The changes in the optical spectra coincide with the observation of an equivalence point at pH 5 in the HCl titration of PTI–LiBr dispersions (Figure S8). The p*K*_b_ extracted from the titration is p*K*_b_=6.4. The p*K*_a_ value associated with the conjugated acid is 7.6 (p*K*_a_=14−p*K*_b_). The conjugated acid is weaker than the conjugated acid of melamin (p*K*_a_=5.0).^[47]^ This indicates that the protonated PTI–LiBr structure is better stabilized than the triazine‐containing monomer, presumably due to conjugation across the PTI‐backbone (Figure 3 f). The observed optical states are summarized in Figure 4 and compared to the optical gaps predicted by the DFT calculations.

Photoconductivity experiments using PTI–LiBr were realized on interdigitated indium tin oxide substrates (Figure S9 a,b). The conductivity of the material is 4×10^−10^ S cm^−1^ in the dark and 1.0×10^−9^ S cm^−1^ in the illuminated state (at 375 nm and an irradiance of 46 mW cm^−2^). Photoconductors using PTI–LiBr undergo changes over the course of the measurement because of the migration of ions. Therefore, the observed hysteresis in IV‐sweeps is dependent on the scan speed of the sweep (Figure S9 c). Materials obtained at 600 °C show no photoresponse and can be considered insulating. This is an important observation since it confirms that an increase in structural order and homogeneity in low‐temperature PTI samples (as seen in PXRD and FT‐IR) and the simultaneous reduction of carbonaceous contaminations translate into better performance of the semiconductor.

**Figure 5:**
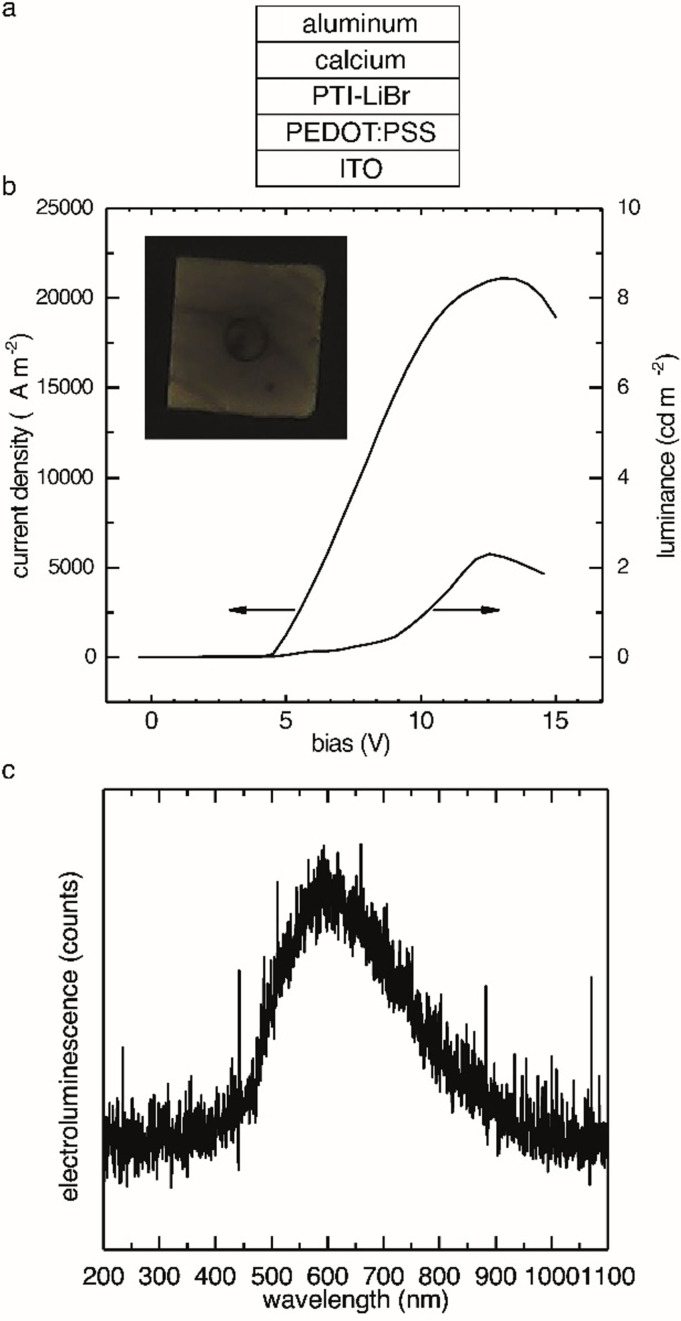
Characterization of a single‐layer OLED with Ca/Al as the cathode, PEDOT:PSS/ITO anode, and PTI‐LiBr as the active material. a) OLED architecture. b) Current density and luminance versus bias with picture of a PTI–LiBr OLED pixel. c) Electroluminescence of the PTI–LiBr OLED.

We have seen so far that (i) PTI‐samples obtained at low temperature conditions (550 °C, 48 h) showed enhanced electrical and optical properties and that (ii) we were able to process PTI‐materials from dispersions into thin films. We therefore prepared single‐layer light emitting device structures with PEDOT:PSS/ITO as anode and Ca/Al as cathode to investigate PTI–LiBr as electroluminescent active material (Figure 5 a). The OLED stack shows a steep diode‐like onset of the device current and electroluminescence emission at ca. 4.5 V in forward bias direction. At 12 V bias we observed a maximum luminance of 2 cd m^−2^. The device showed a current efficiency of ca. 10^−4^ cd A^−1^ (Figure 5 b). Below onset we also observed a high device leakage current which is attributed to film inhomogeneities and pin holes introduced during the drop‐casting preparation process of the PTI–LiBr layer. Similar to previous observations for CN‐films, the electroluminescence maximum of PTI–LiBr has been red‐shifted by 0.5 eV with respect to the PL maximum (Figure 5 c).^[48]^ Defect levels are a likely explanation for the red‐shift. They may arise from small amounts of carbonaceous contaminants still present in the low‐temperature PTI–LiBr sample or nitrogen vacancies in the PTI–LiBr crystals. Electrons are preferably injected into defect levels below the conduction band while holes are preferably injected into defect states above the valence band or subsequently relax into these energetically favored states, resulting in a red‐shifted emission.^[48]^ The presence of carbonaceous contaminants in the low‐temperature PTI–LiBr sample is corroborated in XPS data that show a contribution in the C−C bond region (Figure 1 b) as well as the still slightly carbon‐rich CN ratio (Table 1). While the device performance of this simple single‐layer device is rather limited, we successfully demonstrate that PTI–LiBr can be used as the emissive active layer in OLEDs. In a next step we will introduce hole and electron transport layers and optimize the PTI–LiBr layer preparation to improve the device performance.

Preliminary data from ultraviolet photoelectron spectroscopy (UPS) measurements revealed that PTI–LiBr samples on ITO substrates have a work function (WF) of 4.3 eV and a hole injection barrier (HIB) of 2.8 eV. The ionization potential (IP) equals 7.1 eV (IP=WF+HIB), similar to molecular CN species with high nitrogen content like melem (7 eV).^[49]^ Approximating the conduction band onset using the optical gap extracted from UV/Vis measurements the Fermi level position is near the conduction band and hence pointing towards a n‐doped character (Figure S10 a). A summary of the electronic levels is depicted in Figure S10 b. More studies on the preparation method (inert, environmental conditions) and the influence of the substrate have to be conducted for a more detailed understanding of the electronic structure of poly(triazine imide) and its derivatives.

## Conclusion

We present an improved synthetic protocol for PTI–LiBr and highlight the detrimental effects of high synthetic temperatures on the optical and electronic properties of the material. We observe that the hitherto reported synthetic conditions that employ temperatures of 600 °C (and above) give rise to partial carbonization of PTI–LiBr. The formation of these carbonaceous contaminants in PTI–LiBr gives rise to low‐energy defect states that deteriorate charge transfer pathways and absorb photoluminescence. We find that protonation of PTI–LiBr happens preferentially at the nitrogen atoms of the triazine (C_3_N_3_) moieties. The absorption band of the PTI‐backbone is split due to the symmetry breaking upon protonation. Protonation also increased the quantum yield of PTI suspensions up to a factor of 9. This indicates a facile route to partial exfoliation of the π‐stacked PTI‐structure in solutions of aqueous acids. Finally, we are able to produce a simple OLED structure with PTI–LiBr as an active, metal‐free material to demonstrate electroluminescence. The maximum of the emission is red‐shifted by 0.5 eV in regard to the photoluminescence. This observation is assigned to the presence of defect levels above valence band and below conduction band. UPS results revealed a high ionization potential (7.1 eV) and hole injection barrier (2.8 eV). This finding reveals a possible route towards optimization of the graphitic carbon nitride OLED by introduction of transport layers reducing the injection barriers. Future use of PTI (and analogous, layered CN‐materials) in optical and electronic applications is contingent on (i) further reduction of contributions arising from defect states and (ii) development of methods to access its crystal interfaces as well as PTI monolayers on suited substrates.

## Conflict of interest

The authors declare no conflict of interest.

## Supporting information

As a service to our authors and readers, this journal provides supporting information supplied by the authors. Such materials are peer reviewed and may be re‐organized for online delivery, but are not copy‐edited or typeset. Technical support issues arising from supporting information (other than missing files) should be addressed to the authors.

Supporting Information
