# Supplementary material for: Optimized Synthesis of Solution‐Processable Crystalline Poly(Triazine Imide) with Minimized Defects for OLED Application
Source: Angew Chem Int Ed Engl. 2021 Dec 18;61(3):e202111749. doi: 10.1002/anie.202111749 (PMC9300060; doi:10.1002/anie.202111749)
Supplement: Supplementary file 1 — Supporting Information [file ANIE-61-0-s001.pdf]

Angewandte Chemie
Eine Zeitschrift der Gesellschaft Deutscher Chemiker

Supporting Information

Optimized Synthesis of Solution-Processable Crystalline Poly(Triazine Imide) with Minimized Defects for OLED Application

*David Burmeister, Ha Anh Tran, Johannes Müller, Michele Guerrini, Caterina Cocchi, Julian Plaickner, Zdravko Kochovski, Emil J. W. List-Kratochvil, and Michael J. Bojdys**

anie_202111749_sm_miscellaneous_information.pdf

Experimental

Synthesis of PTI-LiBr: 1 g of precursor dicyandiamide (DCDA, Sigma Aldrich >99%) is ground with a vacuum dried eutectic salt mixture of LiBr and KBr (Sigma Aldrich /Acros Organics >99%) (15 g; 52:48 wt%, m.p. 348°C) in a glovebox. The reaction mixture is filled into a quartz ampule and sealed. The sealed quartz ampule is placed vertically into a furnace (Nabertherm, L 5/11/B180, 2.4 kW) at 400 °C for 4 h. Then the temperature is increased (10 K/min) to the final condensation temperature for the desired timeframe. The ampule is removed at room temperature, opened, and the salt block is dissolved in dest. Water in a 50 mL Falcon. The slurry is centrifuged and the supernatant is decanted. The pellet is re-dispersed in hot water on a shaker and centrifuged again. The supernatant is decanted and the process is repeated for two times with hot water and two times with methanol (>99% for synthesis). The resulting pellet is redispersed in methanol and the methanol is evaporated. The resulting powder is dried under vacuum at 200 °C for 24 h. The furnace geometry and temperature homogeneity play a role for the synthesis of organic materials at temperatures close to carbonization. For repeatable experiments the ampule should be placed at the same spot in the furnace.

PTI-IF was obtained following the procedure proposed by Suter et al. (https://doi.org/10.1039/C8SC05232H) using PTI-LiBr synthesized at 550 °C for 48 h.

First-principle calculations: First-principles calculations are performed on model monolayer structures featuring various protonation conditions – see Figure S6. The unit cell of the PTI monolayer is formed by 18 atoms which comprise the two triazine rings and three imide bridges atoms and the system is assumed to be periodic only in the planar directions. 10 Å of vacuum are included in the directions perpendicular to the layer to avoid unphysical interactions between the replica. Protonation is realized by placing one dissociated HCl molecule per unit cell in proximity to the protonation site (nitrogen at one triazine ring or nitrogen at one imide-bridge, see Figure S6a) and then relaxing the system. The explicit inclusion of the Cl counterion ensures charge neutrality and includes the electrostatic effect of the anion on the protonated system. The equilibrium position of the Cl^- ion, relative to the protonated backbone, is obtained with a further structural optimization of the whole system that places it at the pore centre as shown in Figure S6b-c.

Ground state calculations are performed from spin-restricted DFT [Hohenberg P and Kohn W Phys. Rev. 136 B864 (1964) and Kohn W and Sham L J Phys. Rev. 140 A1133 (1965)] as implemented in the pseudopotential, plane-wave code Quantum Espresso (QE). A uniform 6x6x1 k-mesh is adopted to sample the Brillouin zone, the PBE functional [REF Perdew J P, Burke K and Ernzerhof M Phys. Rev. Lett. 77 3865 (1996)] is used to approximate the exchange-correlation potential, optimized norm conserving pseudopotentials are employed with 50 Ry (200 Ry) plane-wave cut-off to represent the wavefunctions (charge density). The pairwise Tkatchenko-Scheffler scheme [A. Tkatchenko and M. Scheffler, Phys. Rev. Lett. 102, 073005 (2009)] is adopted to include dispersion interactions. The structures are optimized without imposing any symmetry constraint until residual interatomic forces are smaller than 10^{-5} Ry/Bohr. Optical absorption spectra reported are computed from time-dependent DFT [Runge and Gross Physical Review Letters. 52 (12): 997–1000 (1984)] in the linear-response approach. For these calculations the turbo-TDDFT routine of QE is used, implementing the Liouville−Lanczos algorithm. A Lorentzian of 130 meV is applied to each peak.

Preparation of PTI-LiBr dispersions: Sonication of Flakes was conducted with a BANDELIN HD 2200/-U (200 W, HF 20 kHz) sonotrode setup with a MS72 sonotrode with the amplitude set to 10% in continuous mode. 30 mg of PTI-LiBr and 5 mL dist. water are added into a 50 mL falcon tube. The falcon tube is placed in an ice bath and the sonotrode has been immersed about 3-5 mm into the dispersion. Dispersions are sonicated for 3 h, centrifuged at 7690 g for 1 min to reduce large particles and the supernatant was removed from the pellet with a pipet into a falcon. Dispersions with different HCl concentration for optical experiments were prepared by adding 50 µL of the PTI-LiBr dispersion to 3 mL of a corresponding HCl dilution.

Preparation of Photoconductor devices: Ossila "Interdigitated ITO Substrates for OFET and Sensing" S161: $w \times L$: 30 mm × 50 µm were coated with a PTI-LiBr dispersion by drop-casting.

Photoconductor characterisation: Thorlabs LED 375 nm has been controlled by a Keithley 2461 in pulse mode at 3.6 V and a current limit of 1 A. The pulse chain was set to 1 s bias and 5 s off repeating ten times. The interdigitated substrate was contacted by an ossila board "Push-Fit Test Board for Photovoltaic Substrates (8 pixel)" and IV characterisation was conducted with a source measure unit (4200A SCS parameter analyser; Keithley). **For Figure S9a** the LED was focused with a Thorlabs collimator adapter (SM1U25) on the substrate and the irradiance was determined with a thorlabs power meter (PM120VA) to be 458 Wm^{-2}. The current without 375 nm irradiation was determined to be 0.050 µA, with irradiation the current was 0.122 µA at a film thickness of 100 µm and 20 V applied.

$$\sigma = \frac{1}{\rho} = \frac{L}{\frac{U}{I} A} = \frac{L}{\frac{U}{I} w\, t}$$

ρ= electrical resistivity
σ=electrical conductivity
U=bias
I=current
A=surface area
w=channel width
t=film thickness
L=channel length

For **Figure S9b** the LED was not focused with a collimator and the irradiance was determined by calculating it in dependence on the distance between substrate and LED light source.

Film thickness measurements: For extraction of conductivity film thickness values have been obtained with an Olympus LEXT laser scanning microscope.

Fourier transform infrared (FT-IR): Spectra were recorded from solid on a Thermo Scientific Nicolet iS5 spectrometer (Thermo Fisher Scientific, Waltham, MA, USA) in the wavenumber range of 4000-600 cm-1 with resolution of 4 cm-1.

Raman: UV Raman spectra were recorded with a Horiba T64000 spectrometer in single-grating mode. The excitation source was provided by a diode-pumped solid-state laser from CryLas at a wavelength of 266 nm and power of 4 mW. The light was focused on the sample with a Thorlabs LMU-40x-UVB objective (backscattering geometry). A notch filter with cut-off at 220 cm^{-1} was used to filter out the elastically scattered light. The acquired Raman spectra were calibrated by comparison with the spectrum of a Ga_2O_3 crystal by using a quadratic calibration curve. This procedure allows for reduction of the experimental error at approximately 5 cm^{-1}, which is below the spectral resolution of 8 cm^{-1}.

Powder X-ray: Structural analysis of the prepared PTI-MX was performed with a Bruker D2 Phaser X-Ray powder diffractometer (XRD) in Bragg-Brentano geometry. X-rays were generated by a Cu $K_{\alpha1+2}$ source at 30 kV operating voltage and collected with a LynxEye detector.

Photoluminescence, Photoluminescence excitation, quantum yield, Lifetime: Edinburgh Instruments FLS 980 spectrometer. Photoluminescence, photoluminescence excitation and quantum yield were measured using a Xe lamp. The quantum yield was determined in a direct excitation set up with an integrating sphere. Films were drop-casted from ethanol dispersions on quartz substrates PGO 10x10x0.5 mm. Further, it is important to note that the presented samples were prepared and characterised on the same day. Diffusion of protons in PTI-LiBr has not been studied yet. It is possible that crystals of PTI-LiBr are not fully protonated because the system has had not enough time to equilibrate. This might explain why the luminescence of the basic Li-defect is still present in acidic solutions. Lifetime measurements were conducted with an Edinburgh Instruments 375 nm pulsed laser set to a 50 ns pulse period. Evaluation was conducted with the instrument software choosing a two exponential decay function.

UV-Vis: UV-VIS-measurements were performed in ambient conditions with a PerkinElmer Lambda 950 spectrometer in standard transmission operation, 1 nm step size. Films were measured on 10x10x0.5 mm quartz substrates. Dispersions were measured in a quartz cuvette with 1 cm optical path.

XPS, UPS: JOEL JPS-9030 Photoelectron Spectrometer with an Al kα (1486 eV) excitation source and a monochromator. Quantitative comparison of nitrogen and carbon in single spectra were performed by signal integration after background subtraction of a Shirley function. The samples were prepared by drop-casting PTI dispersions (MeOH) on ITO coated glass substrates to minimize charging and to avoid background carbon signals from e.g. carbon tape as substrate. For UPS experiments, a hydrogen discharge lamp (employing H Lyman-α lamp from Excitech, 10.2 eV) was used.(doi: 10.1016/j.orgel.2016.11.032) For determination of the secondary electron cut off (SECO) a negative bias of 10 V was applied to clear the analyser work function. All measurements were performed at room temperature. The obtained values were rounded to one decimal place. The work function was determined by linear fitting of the background and the linear region of the SECO, and reading of the point of intersection. The hole injection barrier was determined by linear fitting of the background and the valence band onset, and reading of the point of intersection. The error of the measurement is ±0.1 eV. Samples for UPS were prepared by dispersing 6 mg/mL of PTI-LiBr in MeOH (Roth, ≥99%) by sonication for 10 min and subsequently dropcasting 50 µL of the dispersion at 60 °C on ITO coated glas substrates (1x1cm).

ssNMR: Cross polarization magic-angle spinning (CP-MAS) solid-state NMR spectra were recorded on a Bruker Avance 400 MHz spectrometer operating at 100.6 MHz (^{13}C).

SEM: The sample was dispersed in Methanol (~0.6 mg/mL) with a micropipette and 0.5-1 µL were dropcast in the middle of a holy carbon TEM grid. SEM images have been recorded with a GeminiSEM 500 electron microscope (Carl Zeiss GmbH, Germany).

Low-dose, high-resolution transmission electron microscopy: The sample was dispersed in Methanol (~0.6 mg/mL) with a micropipette and 0.5-1 µL were dropcast in the middle of a holy carbon TEM grid. The TEM grids were loaded into a cryogenic transfer holder (Gatan 914, Gatan, Munich, Germany) at room temperature and transferred to the TEM. Once in the TEM, the holder was cooled down with liquid nitrogen and imaging was performed with a low dose acquisition scheme using SerialEM (doi: 10.1016/j.jsb.2005.07.007) on JEM-2100 (JEOL GmbH, Eching, Germany) operated at 200 kV and equipped with a 4 k × 4 k CMOS digital camera (TVIPS TemCam-F416). HRTEM images were acquired at a magnification of 500,000×, corresponding to a pixel size of 0.23 Å at the specimen level, while keeping the total electron dose below 20 e Å^{-2}. All imaging was carried out at temperatures around 90 K.

PTI-LiBr HCl titration: 26 mg PTI-LiBr were suspended in 30 mL dest. water by sonication. The pH value changed from 5.5 to 9.4 after suspension of PTI-LiBr. A 0.1 M HCl solution was used as titrant and added in 50 µL then 100 µL steps while stirring with a magnet stirrer. The pH was monitored with a pH glas electrode. The experiment was conducted at room temperature.PTI-LiBr dispersion for OLED preparation: 30 mg of PTI-LiBr and 5 mL chlorobenzene (Sigma-Aldrich, anhydrous, 99.8%) are added into a 50 mL falcon tube. The falcon tube is placed in an ice bath and the sonotrode has been immersed about 3-5 mm into the dispersion. Dispersions are sonicated for 3 h, centrifuged at 95 g for 5 min. The supernatant is transferred into a new falcon tube and the procedure is repeated at 857 g for 5 min two times. Sediments were re-dispersed via sonication (10 min) in a sonication bath prior to usage.

OLED preparation: ITO-coated glass substrates (sheet resistance = 20 Ω per square) were cleaned by sequential sonication (10 minutes) in (i) acetone and (ii) isopropanol followed by drying *via* a nitrogen gun. The substrates were then treated *via* O_2 plasma (partial pressure 1.2 × 10$^{−1}$ mbar) for 15 minutes at 10.2 W. 50 nm PEDOT:PSS (Osilla) films were spin coated as hole injection layer and heated to 220 °C for 10 min. 40 µL PTI-LiBr dispersion were drop cast at 60 C from the chlorobenzene dispersion onto the pixel areas of the PEDOT:PSS covered substrate (ca. 150 mm^2). 5 nm Calcium and 200 nm Aluminium were evaporated in a PVD chamber at 10^{-5} mbar. Finally the OLEDs were encapsulated with UV-curable resin (Osilla) and a glass slide. Current density–voltage–luminance characterization was performed with a Keithley 2612B source meter and a Konica Minolta LS-160 luminance meter in a purpose-built setup. Electroluminescence spectra were taken with a CS2000 spectrometer (Ocean Optics) using OceanView software.

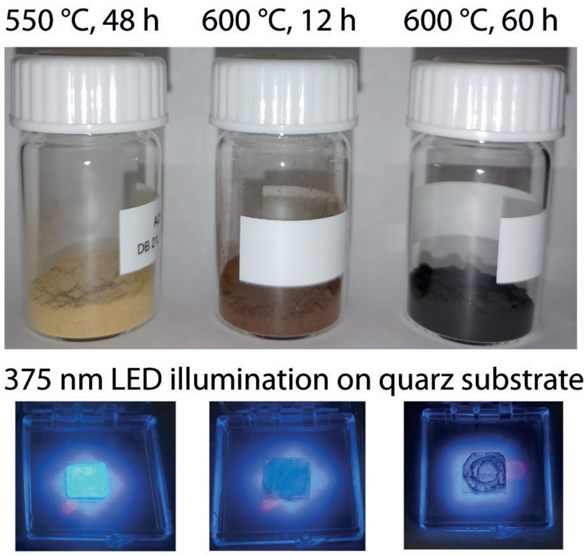

Figure S1. Three vials with the product of the condensation reaction at 550 °C, 48 h, 600 °C, 12h and 600 °C, 72 h are depicted. Underneath are the corresponding films on quartz substrates drop casted from dispersion for the 550 °C 48 h product, 600 °C 12 h and 600 °C 72 h product. The brown color is a result of on setting carbonization and leads to self-absorption and deteriorates charge transport through the polycrystalline material.

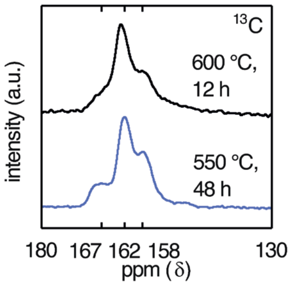

Fig**ure S2**.Solid state NMR (ssNMR) of PTI-LiBr obtained from conditions reported in literature 600 °C 12 h and at lower temperature 550 °C, 48 h. The spectrum obtained from the product synthesized at lower temperature has sharper bands. The three observed bands correspond to three carbon environments present in PTI-LiBr as well as PTI-LiCl at 167.0 ppm 162.0 ppm and 158.0 ppm.

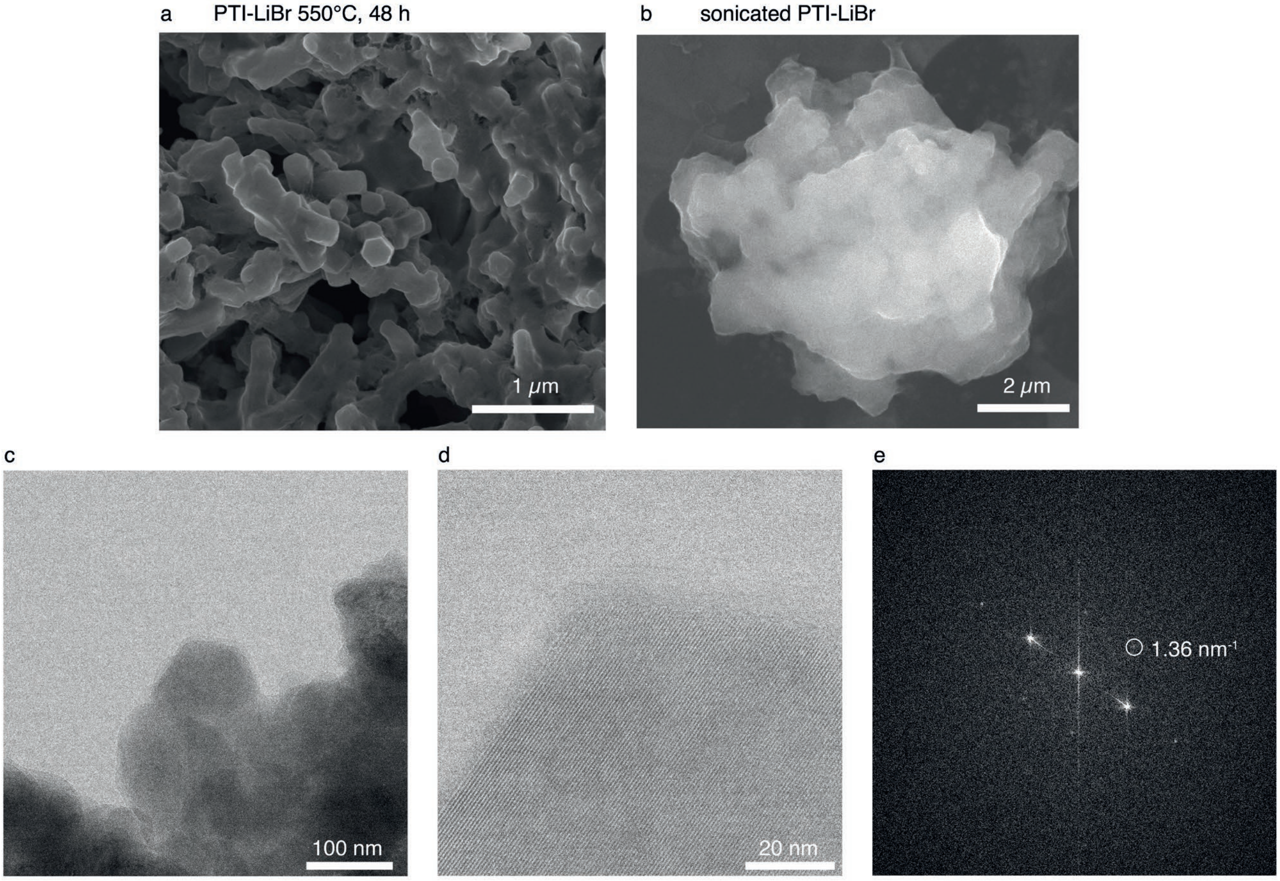

Figure S3. SEM and HR-TEM studies of PTI-LiBr product. a) The anticipated hexagonal platelets of PTI-LiBr (5 kV acceleration voltage). b) PTI-LiBr product after 30 min sonication (20 kV acceleration voltage). SEM pictures after sonication show typically a film of debris covering all structures. HR-TEM at an acceleration Voltage of 200 kV of PTI-LiBr from 550 °C synthesis. c) 100.000x magnification HR-TEM image d) 500.000x magnification HR-TEM image e) Fourier transformation of d) reveals the characteristic 100 reflex at 1.36 nm^{-1} (0.73 nm) and the hexagonal periodicity of PTI-LiBr.

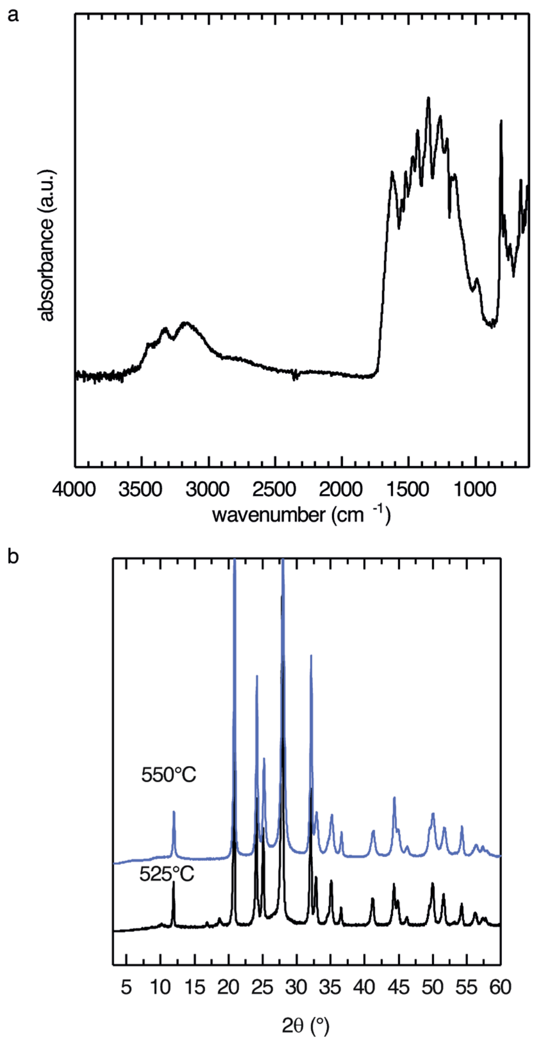

Figure S4. a) FT-IR of PTI-LiBr product from 525 °C for 48 h b) PXRD of PTI-LiBr product from 525 °C for 48 h compared to 550 °C for 48h. Additional contributions in the NH region and additional PXRD peaks indicate incomplete condensation.

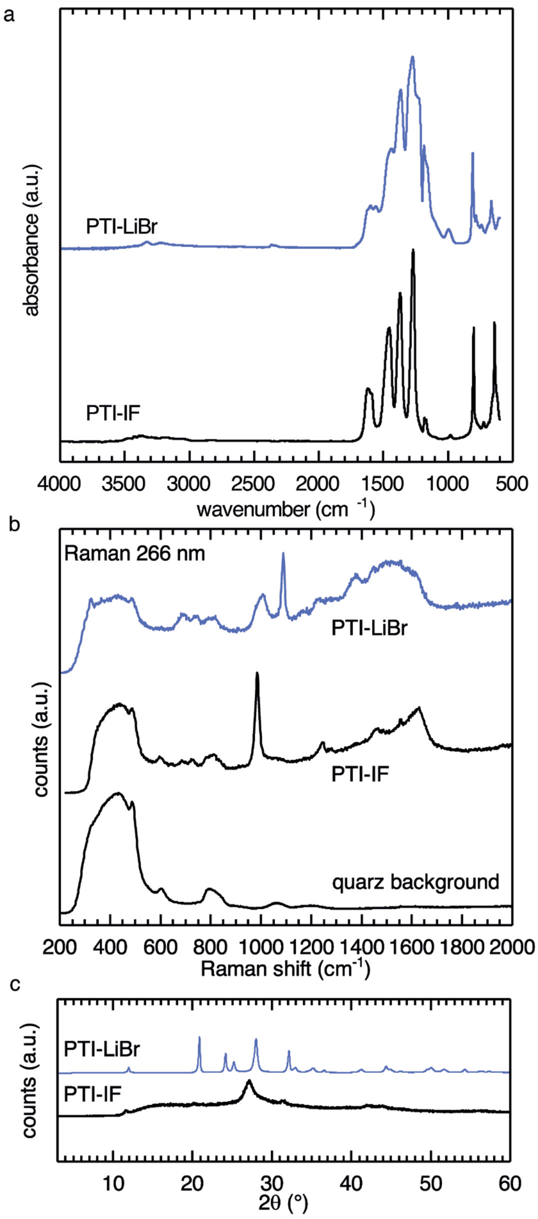

Figure S5. a) FT-IR b) UV-Raman and c) PXRD of PTI-LiBr and PTI-IF. De-intercalation of PTI-LiBr results in more defined IR and Raman bands. PTI-LiBr has an unexpected strong Raman signal at 1088 cm^{-1}. This could be due to formation of small amounts of lithium carbonate in a surface reaction. The lithium defect can undergo hydrolysis in presence of atmospheric water. The reaction of LiOH with atmospheric CO_2 results in lithium carbonate. The PXRD of PTI-IF shows the loss of the graphitic order after removing the structure directing salt ions.

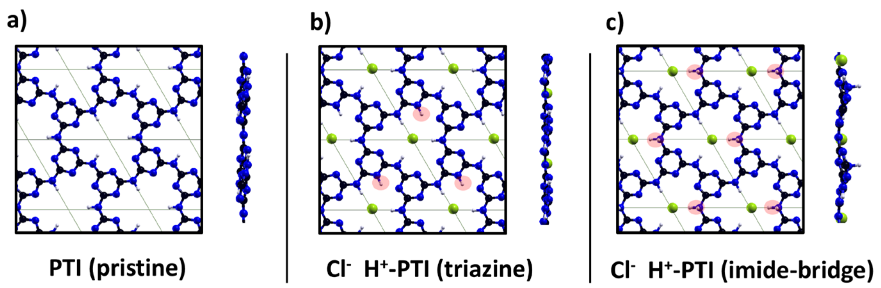

Figure S6. PTI single monolayer relaxed structures in-vacuo, relative to PTI-IF (a), the triazine-protonated (b) and the imide-bridge protonated (c) backbones. In the protonated structures we have also reported the formation energies per unit-cell volume evaluated as the difference between the total energy of the protonated system and the sum of the energies of the separated pristine monolayer and HCl subsystems.

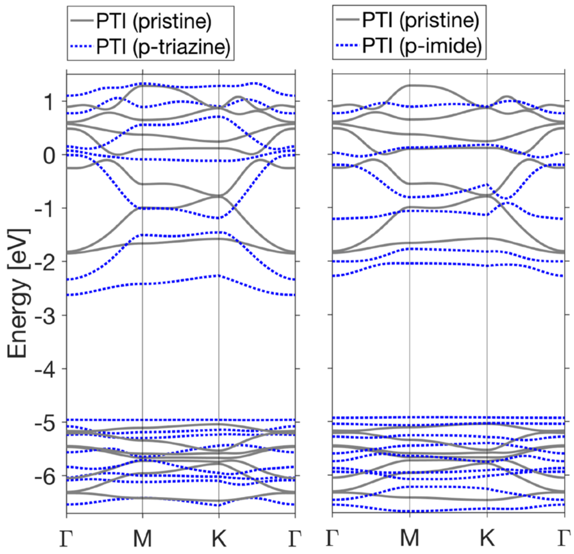

Figure S7. Electronic bandstructures of pristine PTI-IF (gray lines) and triazine / imide-bridge protonated-PTI (blue dashed lines).

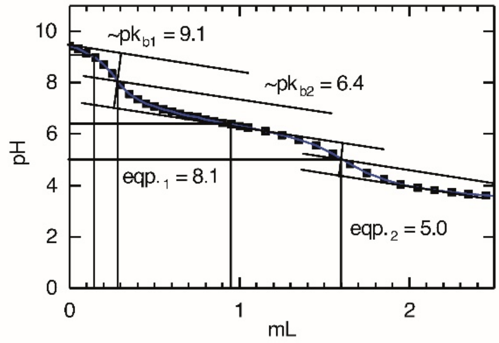

Figure S8. HCl (0.1 M) titration of 26 mg PTI-LiBr (550 °C, 48 h) dispersion. The second equivalence point at pH 5 corresponds to the pH region where also changes in the optical spectra take place (emergence of 330 nm, 420 nm photoluminescence and 260 nm absorption band).

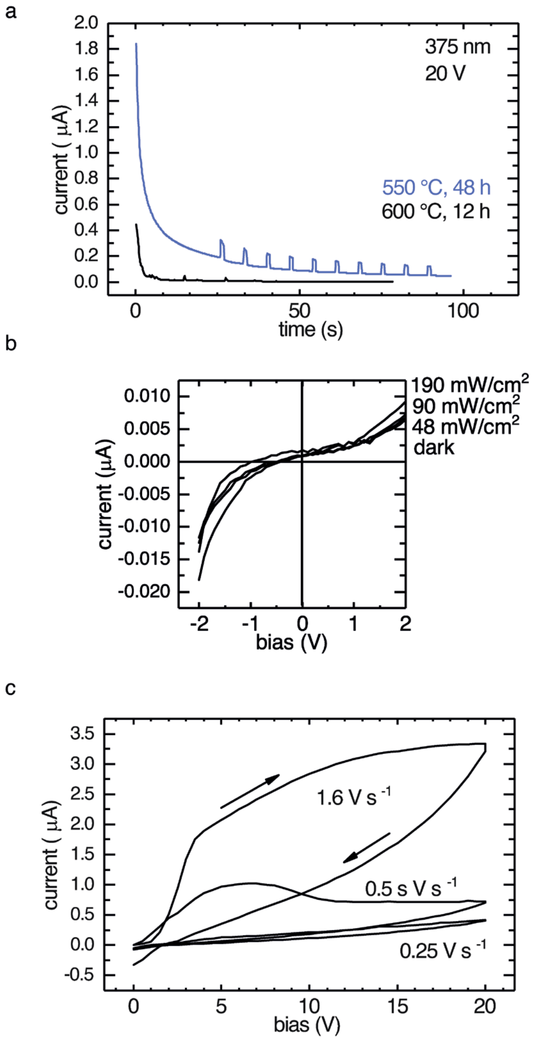

Figure S9. a) Comparison of photoconductor employing PTI-LiBr from 600 °C and 550 °C. Charge transport and photocurrent are absent in the 600 °C material due to the observed partial carbonization (black line). In the device employing PTI-LiBr from 550 °C current is able to cross the channel of the interdigitated substrate and photocurrent is observed when excited with a 375 nm LED at 375 nm at an irradiance of 46 mW cm^{-2}. b) Irradiance dependent IV sweep of photoconductor device. Increased irradiance with a 375 nm LED results in increased current flow. The low current is result of the polycrystalline material property (grain boundaries) and likely energetic disorder at partially intercalation free layers. b) IV sweeps of an interdigitated device employing 550 °C, 48 h product with different sweep speed. The observed hysteresis is due to migration of Li^+ and Br^- ions.

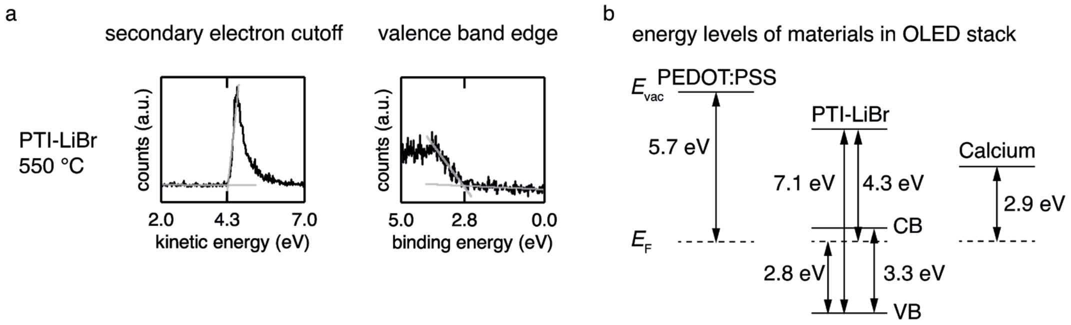

Figure S10. a) Ultraviolet photoelectron spectroscopy (UPS) PTI-LiBr dropcasted on ITO substrates. b) Energy levels of OLED materials. Work functions of PEDOT:PSS and calcium have been extracted from literature (doi: 10.1063/1.2435350, doi: 10.1063/1.32353). The valence band of PTI-LiBr is measured by UPS while the conduction band is estimated combining the latter with the UV-Vis spectroscopy data.

Experimenteller Teil

Synthese von PTI-LiBr: 1 g der Vorstufe Dicyandiamid (DCDA, Sigma Aldrich >99%) wird mit einer vakuumgetrockneten eutektischen Salzmischung aus LiBr und KBr (Sigma Aldrich /Acros Organics >99%) (15 g; 52:48 Gew.-%, m.p. 348°C) in einer Glovebox vermahlen. Die Reaktionsmischung wird in eine Quarzampulle gefüllt und verschlossen. Die versiegelte Quarzampulle wird für 4 h senkrecht in einen Ofen (Nabertherm, L 5/11/B180, 2,4 kW) bei 400 °C gestellt. Anschließend wird die Temperatur für die gewünschte Zeitspanne (10 K/min) bis zur endgültigen Kondensationstemperatur erhöht. Die Ampulle wird bei Raumtemperatur entnommen, geöffnet und der Salzblock wird in dest. Wasser in einem 50 mL Falcon gelöst. Die Aufschlämmung wird zentrifugiert und der Überstand wird dekantiert. Das Pellet wird erneut in heißem Wasser auf einem Schüttler dispergiert und erneut zentrifugiert. Der Überstand wird dekantiert und der Vorgang wird zweimal mit heißem Wasser und zweimal mit Methanol (>99% für die Synthese) wiederholt. Das resultierende Pellet wird erneut in Methanol dispergiert und das Methanol verdampft. Das so entstandene Pulver wird 24 Stunden lang unter Vakuum bei 200 °C getrocknet. Die Ofengeometrie und die Temperaturhomogenität spielen eine Rolle für die Synthese organischer Materialien bei Temperaturen nahe der Karbonisierung. Für wiederholbare Experimente sollte die Ampulle an der gleichen Stelle im Ofen platziert werden.

PTI-IF wurde nach dem von Suter et al. (https://doi.org/10.1039/C8SC05232H) vorgeschlagenen Verfahren unter Verwendung von PTI-LiBr gewonnen, das 48 Stunden lang bei 550 °C synthetisiert wurde.

Ab initio Berechnungen: Berechnungen werden an Modell-Monolagen-Strukturen mit verschiedenen Protonierungsbedingungen durchgeführt - siehe Abbildung S6. Die Einheitszelle der PTI-Monolage besteht aus 18 Atomen, die die beiden Triazinringe und drei Imidbrückenatome umfassen, und es wird angenommen, dass das System nur in den planaren Richtungen periodisch ist. In den Richtungen senkrecht zur Schicht sind 10 Å Vakuum eingeschlossen, um nicht-physikalische Wechselwirkungen zwischen den Nachbildungen zu vermeiden. Die Protonierung wird durch Platzierung eines dissoziierten HCl-Moleküls pro Einheitszelle in der Nähe der Protonierungsstelle (Stickstoff an einem Triazinring oder Stickstoff an einer Imidbrücke, siehe Abbildung S6a) und anschließende Entspannung des Systems realisiert. Durch die ausdrückliche Einbeziehung des Cl-Gegenions wird die Ladungsneutralität gewährleistet und die elektrostatische Wirkung des Anions auf das protonierte System berücksichtigt. Die Gleichgewichtsposition des Cl-Ions in Bezug auf das protonierte Rückgrat wird durch eine weitere strukturelle Optimierung des gesamten Systems erreicht, bei der es im Porenzentrum platziert wird, wie in Abbildung S6b-c dargestellt.

Grundzustandsberechnungen werden auf der Grundlage der spinbeschränkten DFT [Hohenberg P und Kohn W Phys. Rev. 136 B864 (1964) und Kohn W und Sham L J Phys. Rev. 140 A1133 (1965)] durchgeführt, wie sie im pseudopotential, ebenen Wellencode Quantum Espresso (QE) implementiert ist. Für die Abtastung der Brillouin-Zone wird ein gleichmäßiges 6x6x1 k-Gitter verwendet, das PBE-Funktional [REF Perdew J P, Burke K und Ernzerhof M Phys. Rev. Lett. 77 3865 (1996)] wird zur Annäherung des Austausch-Korrelations-Potentials verwendet, und es werden optimierte norm-erhaltende Pseudopotentiale mit 50 Ry (200 Ry) Ebenen-Wellen-Cut-Off zur Darstellung der Wellenfunktionen (Ladungsdichte) eingesetzt. Das paarweise Tkatchenko-Scheffler-Schema [A. Tkatchenko und M. Scheffler, Phys. Rev. Lett. 102, 073005 (2009)] wird verwendet, um Dispersionswechselwirkungen zu berücksichtigen. Die Strukturen werden ohne jegliche Symmetriebeschränkung optimiert, bis die verbleibenden interatomaren Kräfte kleiner als 10^{-5} Ry/Bohr sind. Die angegebenen optischen Absorptionsspektren werden aus zeitabhängigen DFT-Berechnungen [Runge und Gross Physical Review Letters. 52 (12): 997-1000 (1984)] mit dem Linear-Response-Ansatz berechnet. Für diese Berechnungen wird die Turbo-TDDFT-Routine von QE verwendet, die den Liouville-Lanczos-Algorithmus implementiert. Ein Lorentzian von 130 meV wird auf jeden Peak angewendet.

Herstellung von PTI-LiBr-Dispersionen: Die Ultraschall Behandlung der PTI dispersionen wurde mit einer BANDELIN HD 2200/-U (200 W, HF 20 kHz) Sonotrodenanordnung mit einer MS72-Sonotrode durchgeführt, wobei die Amplitude im kontinuierlichen Modus auf 10 % eingestellt wurde. 30 mg PTI-LiBr und 5 mL dest. Wasser werden in ein 50 mL Falcon Tube gegeben. Das Falcon Tube wird in ein Eisbad gestellt, und die Sonotrode wird etwa 3-5 mm in die Dispersion eingetaucht. Die Dispersionen werden 3 Stunden lang beschallt, dann 1 Minute lang bei 7690 g zentrifugiert, um große Partikel zu entfernen, und der Überstand wird mit einer Pipette in ein Falcon-Röhrchen abgenommen. Dispersionen mit unterschiedlichen HCl-Konzentrationen für optische Experimente wurden durch Zugabe von 50 µl der PTI-LiBr-Dispersion zu 3 mL einer entsprechenden HCl-Verdünnung hergestellt.

Vorbereitung von Fotoleiterbauteilen: Ossila "Interdigitated ITO Substrates for OFET and Sensing" S161: b × L: 30 mm × 50 µm wurden im Tropfengießverfahren mit einer PTI-LiBr-Dispersion beschichtet.

Charakterisierung des Photoleiters: Die Thorlabs LED 375 nm wurde von einem Keithley 2461 im Pulsmodus bei 3,6 V und einer Strombegrenzung von 1 A gesteuert. Die Pulskette wurde auf 1 s Spannung und 5 s Pause eingestellt und zehnmal wiederholt. Das

interdigitale Substrat wurde mit einer Ossila-Platte "Push-Fit Test Board for Photovoltaic Substrates (8 pixel)" kontaktiert, und die IV-Charakterisierung wurde mit einem Quellenmessgerät (4200A SCS parameter analyser; Keithley) durchgeführt. Für Abbildung S9a wurde die LED mit einem Thorlabs-Kollimatoradapter (SM1U25) auf das Substrat fokussiert, und die Bestrahlungsstärke wurde mit einem Thorlabs-Leistungsmesser (PM120VA) auf 458 Wm^{-2} bestimmt. Der Strom ohne 375-nm-Bestrahlung wurde mit 0,050 µA bestimmt, mit Bestrahlung betrug der Strom 0,122 µA bei einer Schichtdicke von 100 µm und 20 V angelegt. Die Leitfähigkeit wurde über folgende Beziehung bestimmt:

$$\sigma = \frac{1}{\rho} = \frac{L}{\frac{U}{I}A} = \frac{L}{\frac{U}{I}w\,t}$$

ρ= elektrischer spezifischer Wiederstand
σ=elektrische Leitfähigkeit
U=Vorspannung
I=Strom
A=Oberfläche
w=Kanalbreite
t=Schichtdicke
L=Kanallänge

Für Abbildung S9b wurde die LED nicht mit einem Kollimator fokussiert, und die Bestrahlungsstärke wurde durch Berechnung in Abhängigkeit vom Abstand zwischen Substrat und LED-Lichtquelle ermittelt.

Messungen der Schichtdicke: Zur Ermittlung der Leitfähigkeit wurden die Schichtdicken mit einem Olympus LEXT Laser-Scanning-Mikroskop gemessen.

Fourier-Transformations-Infrarot (FT-IR): Die Spektren wurden von Feststoffen mit einem Thermo Scientific Nicolet iS5 Spektrometer (Thermo Fisher Scientific, Waltham, MA, USA) im Wellenzahlbereich von 4000-600 cm-1 mit einer Auflösung von 4 cm^{-1} aufgenommen.

Raman: UV-Raman-Spektren wurden mit einem Horiba T64000-Spektrometer im Einzelgittermodus aufgenommen. Als Anregungsquelle diente ein diodengepumpter Festkörperlaser von CryLas mit einer Wellenlänge von 266 nm und einer Leistung von 4 mW. Das Licht wurde mit einem Thorlabs LMU-40x-UVB-Objektiv (Rückstreuungsgeometrie) auf die Probe fokussiert. Ein Kerbfilter mit Cut-off bei 220 cm^{-1} wurde verwendet, um das elastisch gestreute Licht herauszufiltern. Die aufgenommenen Raman-Spektren wurden durch Vergleich mit dem Spektrum eines Ga2O3-Kristalls unter Verwendung einer quadratischen Kalibrierungskurve kalibriert. Dieses Verfahren ermöglicht eine Verringerung des experimentellen Fehlers auf etwa 5 cm^{-1}, was unterhalb der spektralen Auflösung von 8 cm^{-1} liegt.

Röntgenbeugung des Pulvers: Die Strukturanalyse des hergestellten PTI-MX wurde mit einem Bruker D2 Phaser Röntgenpulverdiffraktometer (XRD) in Bragg-Brentano-Geometrie durchgeführt. Die Röntgenstrahlen wurden von einer Cu-Kα1+2-Quelle bei einer Betriebsspannung von 30 kV erzeugt und mit einem LynxEye-Detektor detektiert.

Photolumineszenz, Photolumineszenzanregung, Quantenausbeute, Lebenszeit: Spektrometer FLS 980 von Edinburgh Instruments. Photolumineszenz, Photolumineszenzanregung und Quantenausbeute wurden mit einer Xe-Lampe gemessen. Die Quantenausbeute wurde bei direkter Anregung mit einer Ulbricht-Kugel bestimmt. Die Filme wurden aus Ethanoldispersionen auf Quarzsubstraten PGO 10x10x0,5 mm im Tropfengussverfahren hergestellt. Dispersionen wurden hergestellt wie in „Herstellung von PTI-LiBr-Dispersionen“ beschrieben**.** Außerdem ist es wichtig zu erwähnen, dass die vorgestellten Proben am selben Tag hergestellt und charakterisiert wurden. Die Diffusion von Protonen in PTI-LiBr ist noch nicht untersucht worden. Es ist möglich, dass die PTI-LiBr-Kristalle in Dispersion nicht vollständig protoniert sind, weil das System nicht genug Zeit hatte, das Equilibrium zu erreichen. Dies könnte erklären, warum die Lumineszenz des basischen Li-Defekts in sauren Lösungen noch vorhanden ist. Die Lebensdauermessungen wurden mit einem gepulsten 375-nm-Laser von Edinburgh Instruments durchgeführt, der auf eine Pulsdauer von 50 ns eingestellt war. Die Auswertung erfolgte mit der Gerätesoftware, die eine zweiexponentielle Abklingfunktion wählte.

UV-Vis: UV-Vis-Messungen wurden unter Umgebungsbedingungen mit einem Lambda 950-Spektrometer von PerkinElmer im Standardtransmissionsbetrieb mit einer Schrittweite von 1 nm durchgeführt. Die Filme wurden auf 10x10x0,5 mm großen Quarzsubstraten gemessen. Dispersionen wurden in einer Quarzküvette mit 1 cm optischem Weg gemessen.

XPS, UPS: JOEL JPS-9030 Photoelektronenspektrometer mit einer Al kα (1486 eV) Anregungsquelle und einem Monochromator. Der quantitative Vergleich von Stickstoff und Kohlenstoff in Einzelspektren erfolgte durch Signalintegration nach Subtraktion des

Hintergrunds mit Hilfe einer Shirley-Funktion. Die Proben wurden durch Tropfengießen von PTI-Dispersionen (MeOH) auf ITO-beschichtete Glassubstrate hergestellt, um die Aufladung zu minimieren und Hintergrundsignale von Kohlenstoff, z. B. von einem Carbonband als Substrat, zu vermeiden. Für UPS-Experimente wurde eine Wasserstoffentladungslampe (mit einer H Lyman-α-Lampe von Excitech, 10,2 eV) verwendet.(doi: 10.1016/j.orgel.2016.11.032) Für die Bestimmung des Sekundärelektronen cutoff (SECO) wurde eine negative Spannung von 10 V an die probe angelegt. Alle Messungen wurden bei Raumtemperatur durchgeführt. Die erhaltenen Werte wurden auf eine Dezimalstelle gerundet. Die Austrittsarbeit wurde durch lineare Anpassung des Hintergrunds und des linearen Bereichs des SECO sowie durch Ablesen des Schnittpunkts bestimmt. Die Lochinjektionsbarriere wurde durch lineare Anpassung des Hintergrunds und des Beginns des Valenzbandes sowie durch Ablesen des Schnittpunkts bestimmt. Der Fehler der Messung beträgt ±0,1 eV. Die Proben für die USV wurden durch Dispergieren von 6 mg/ml PTI-LiBr in MeOH (Roth, ≥99%) durch 10-minütige Beschallung und anschließendes Tropfengießen von 50 µl der Dispersion bei 60 °C auf ITO-beschichtete Glassubstrate (1x1cm) hergestellt.

ssNMR: Kreuzpolarisations-Magic-Angle-Spinning (CP-MAS) Festkörper-NMR-Spektren wurden mit einem Bruker Avance 400 MHz-Spektrometer bei 100,6 MHz (13C) aufgenommen.

REM: Die Probe wurde mit einer Mikropipette in Methanol (~0,6 mg/ml) dispergiert und 0,5-1 µl wurden in die Mitte eines TEM-Gitters aus löchrigem Kohlenstoff getropft. Die REM-Bilder wurden mit einem GeminiSEM 500 Elektronenmikroskop (Carl Zeiss GmbH, Deutschland) aufgenommen.

Hochauflösende Transmissions-Elektronenmikroskopie mit niedriger Dosis: Die Probe wurde mit einer Mikropipette in Methanol (~0,6 mg/mL) dispergiert und 0,5-1 µL wurden in die Mitte eines TEM-Gitters aus löchrigem Kohlenstoff getropft. Die TEM-Gitter wurden bei Raumtemperatur in einen kryo-Transferhalter (Gatan 914, Gatan, München, Deutschland) geladen und zum TEM transportiert. Im TEM wurde der Halter mit flüssigem Stickstoff gekühlt, und die Bildgebung erfolgte mit einem Niedrigdosis-Aufnahmeschema unter Verwendung von SerialEM (doi: 10.1016/j.jsb.2005.07.007) am JEM-2100 (JEOL GmbH, Eching, Deutschland), das bei 200 kV Beschleunigungsspannung betrieben wird und mit einer 4 k × 4 k CMOS-Digitalkamera (TVIPS TemCam-F416) ausgestattet ist. Die HRTEM-Bilder wurden mit einer Vergrößerung von 500.000× aufgenommen, was einer Pixelgröße von 0,23 Å auf der Probenebene entspricht, wobei die Gesamtelektronendosis unter 20 e $Å^{-2}$ gehalten wurde. Alle Aufnahmen wurden bei Temperaturen um 90 K durchgeführt.

PTI-LiBr HCl Titration: 26 mg PTI-LiBr wurden in 30 mL dest. Wasser durch Beschallung suspendiert. Der pH-Wert änderte sich nach der Suspension von PTI-LiBr von 5,5 auf 9,4. Als Titrant wurde eine 0,1 M HCl-Lösung verwendet, die unter Rühren mit einem Magnetrührer in Schritten von 50 µl und dann 100 µl zugegeben wurde. Der pH-Wert wurde mit einer pH-Glaselektrode überwacht. Das Experiment wurde bei Raumtemperatur durchgeführt.

PTI-LiBr-Dispersion zur Herstellung von OLEDs: 30 mg PTI-LiBr und 5 mL Chlorbenzol (Sigma-Aldrich, wasserfrei, 99,8%) werden in ein 50 mL Falcon-Röhrchen gegeben. Das Falcon Tube wird in ein Eisbad gestellt, und die Sonotrode wird etwa 3-5 mm in die Dispersion eingetaucht. Die Dispersionen werden 3 Stunden lang beschallt und anschließend 5 Minuten lang bei 95 g zentrifugiert. Der Überstand wird in ein neues Falcon-Röhrchen überführt, und der Vorgang wird zweimal bei 857 g für 5 min wiederholt. Die Sedimente wurden vor der Verwendung durch Beschallung (10 min) in einem Beschallungsbad erneut dispergiert.

OLED Fabrikation: ITO-beschichtete Glassubstrate (Schichtwiderstand = 20 Ω pro Quadrat) wurden durch aufeinanderfolgende Beschallung (10 Minuten) in (i) Aceton und (ii) Isopropanol gereinigt und anschließend mit einer Stickstoffpistole getrocknet. Die Substrate wurden dann 15 Minuten lang mit O2-Plasma (Partialdruck 1,2 × 10-1 mbar) bei 10,2 W behandelt. 50 nm PEDOT:PSS (Osilla) wurden als Lochinjektionsschicht aufgeschleudert und 10 Minuten lang auf 220 °C erhitzt. 40 µl PTI-LiBr-Dispersion wurden bei 60 °C aus der Chlorbenzoldispersion auf die Pixelflächen des mit PEDOT:PSS beschichteten Substrats (ca. 150 mm^2) getropft. 5 nm Calcium und 200 nm Aluminium wurden in einer PVD-Kammer bei 10^{-5} mbar aufgedampft. Schließlich wurden die OLEDs mit UV-härtbarem Harz (Osilla) und einem Glasobjektträger verkapselt. Die Stromdichte-Spannungs-Leuchtdichte-Charakterisierung wurde mit einem Keithley 2612B-Quellenmessgerät und einem Konica Minolta LS-160-Leuchtdichtemessgerät in einem speziell angefertigten Aufbau durchgeführt. Elektrolumineszenzspektren wurden mit einem CS2000-Spektrometer (Ocean Optics) unter Verwendung der OceanView-Software aufgenommen.

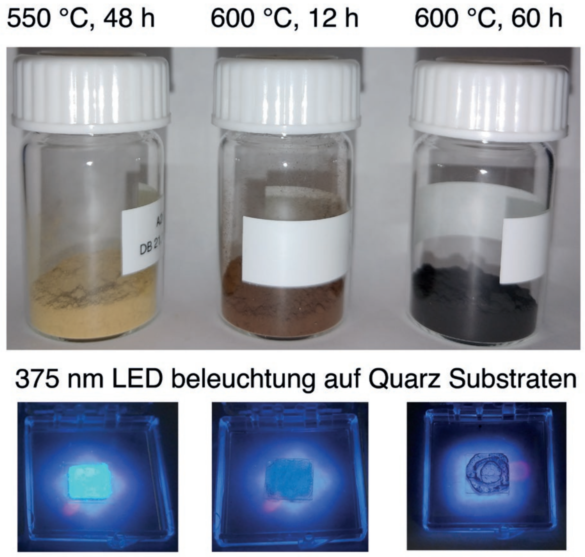

Abbildung S1. Abgebildet sind drei Fläschchen mit dem Produkt der Kondensationsreaktion bei 550 °C, 48 h, 600 °C, 12h und 600 °C, 72 h. Darunter sind die entsprechenden Filme auf Quarzsubstraten zu sehen, die aus einer Dispersion für die Produkte 550 °C, 48 h, 600 °C, 12 h und 600 °C, 72 h im Tropfgussverfahren aufgetragen wurden. Die braune Farbe ist eine Folge der Karbonisierung beim und führt zu Selbstabsorption und verschlechtert den Ladungstransport durch das polykristalline Material.

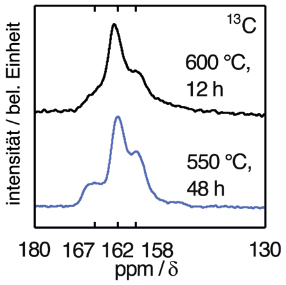

Abbildung S2. Festkörper-NMR (ssNMR) von PTI-LiBr, erhalten unter den in der Literatur angegebenen Bedingungen 600 °C, 12 h, und bei niedrigerer Temperatur 550 °C, 48 h. Das Spektrum des bei niedrigerer Temperatur synthetisierten Produkts weist schärfere Banden auf. Die drei beobachteten Banden entsprechen den drei Kohlenstoffumgebungen in PTI-LiBr analog zu Werten die für PTI-LiCl in der Literatur erwähnt sind bei 167.0 ppm 162.0 ppm und 158.0 ppm.

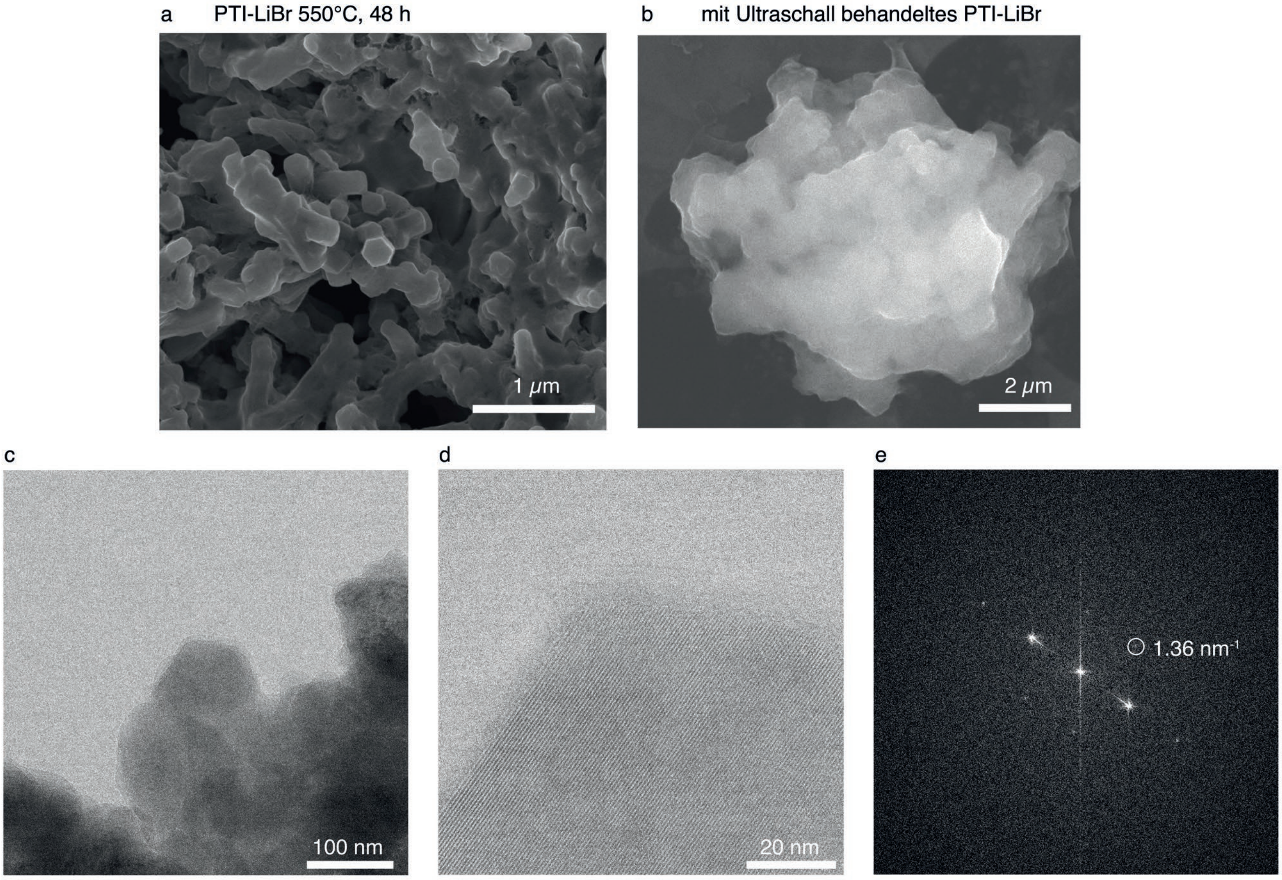

Abbildung S3. REM- und HR-TEM-Untersuchungen des PTI-LiBr-Produkts. a) Die zu erwartenden hexagonalen Plättchen von PTI-LiBr (5 kV Beschleunigungsspannung). b) PTI-LiBr-Produkt nach 30 Minuten Beschallung (20 kV Beschleunigungsspannung). Die REM-Bilder nach der Beschallung zeigen typischerweise einen Film aus Trümmern, der alle Strukturen bedeckt. HR-TEM bei einer Beschleunigungsspannung von 200 kV von PTI-LiBr aus der 550 °C-Synthese. c) HR-TEM-Bild mit 100.000-facher Vergrößerung d) HR-TEM-Bild mit 500.000-facher Vergrößerung e) Die Fourier-Transformation von d) zeigt den charakteristischen 100-Reflex bei 1,36 nm^{-1} (0,73 nm) und die hexagonale Periodizität von PTI-LiBr.

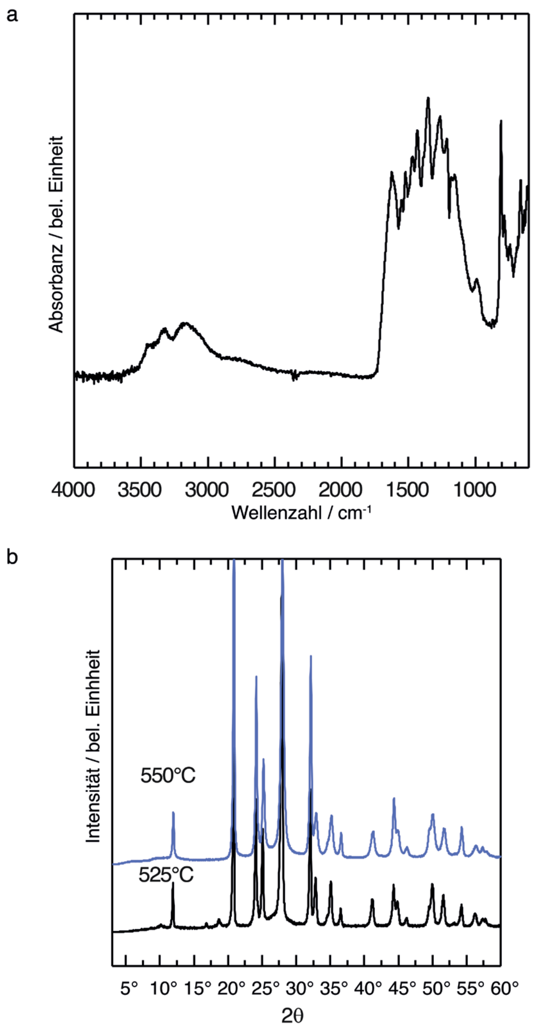

Abbildung S4. a) FT-IR des PTI-LiBr-Produkts bei 525 °C für 48 h b) PXRD des PTI-LiBr-Produkts bei 525 °C für 48 h im Vergleich zu 550 °C für 48 h. Zusätzliche Beiträge im NH-Bereich und zusätzliche PXRD-Peaks weisen auf eine unvollständige Kondensation hin.

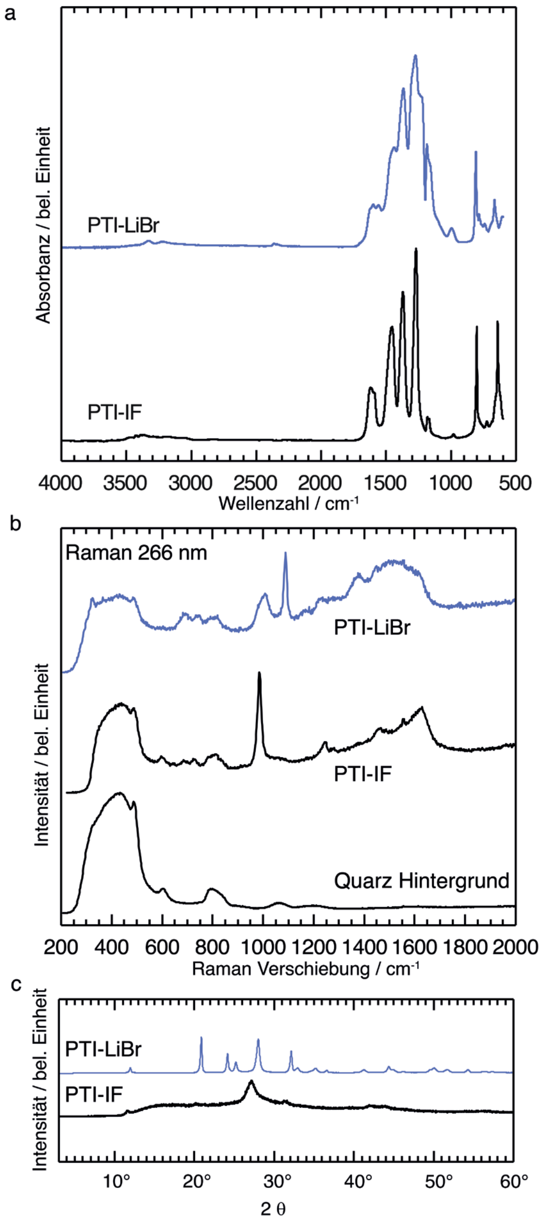

Abbildung S5. a) FT-IR b) UV-Raman und c) PXRD von PTI-LiBr und PTI-IF. Die Deinterkalation von PTI-LiBr führt zu besser definierten IR- und Raman-Banden. PTI-LiBr hat ein unerwartet starkes Ramansignal bei 1088 cm-1. Dies könnte auf die Bildung kleiner Mengen von Lithiumcarbonat in einer Oberflächenreaktion zurückzuführen sein. Der Lithiumdefekt kann in Gegenwart von atmosphärischem Wasser hydrolysiert werden. Die Reaktion von LiOH mit atmosphärischem CO2 führt zur Bildung von Lithiumcarbonat. Das PXRD von PTI-IF zeigt den Verlust der graphitischen Ordnung nach der Entfernung der strukturgebenden Salz-Ionen.

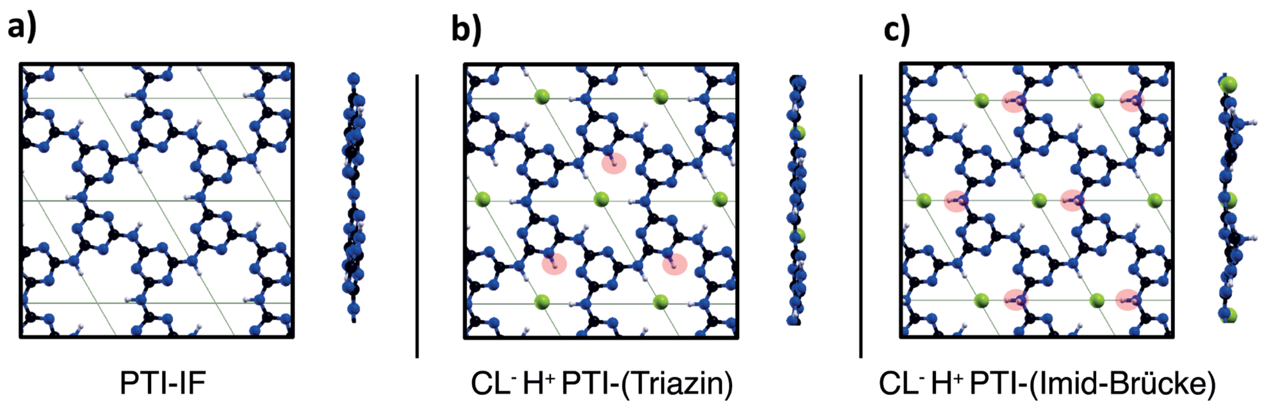

Abbildung S6. Relaxierte PTI-Einzelschicht-Strukturen in-vacuo, bezogen auf PTI-IF (a), das protonierte Triazin (b) und die protonierte Imid-Brücke (c). Für die protonierten Strukturen haben wir auch die Bildungsenergien pro Zellvolumen berechnet, die als Differenz zwischen der Gesamtenergie des protonierten Systems und der Summe der Energien der getrennten ursprünglichen Monoschicht- und HCl-Subsysteme bewertet wurden.

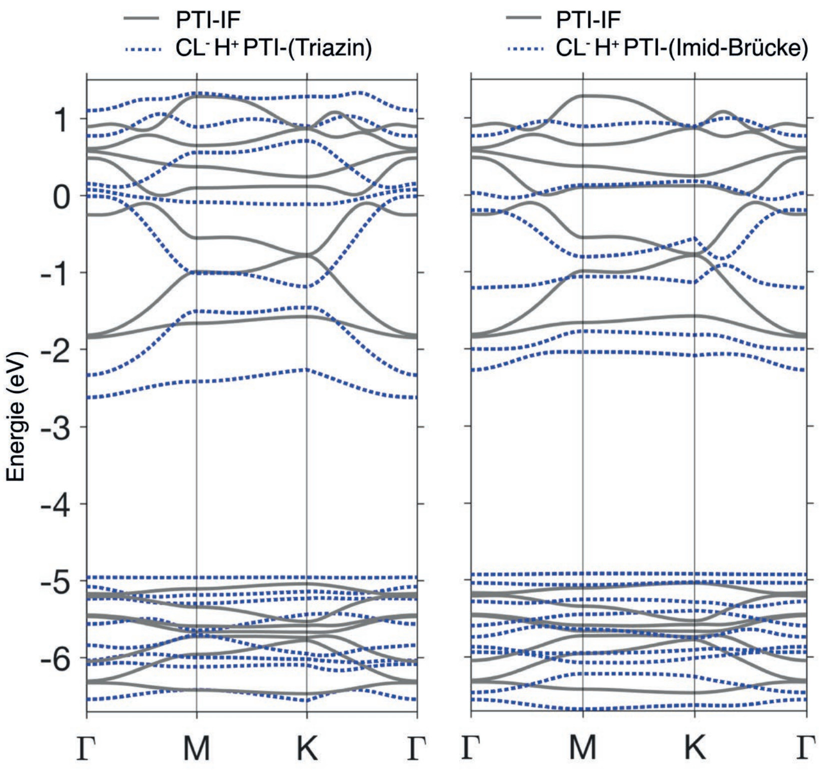

Abbildung S7. Elektronische Bandstrukturen von unbehandeltem PTI-IF (graue Linien) und protoniertem PTI mit Triazin/Imid-Brücke (blaue gestrichelte Linien).

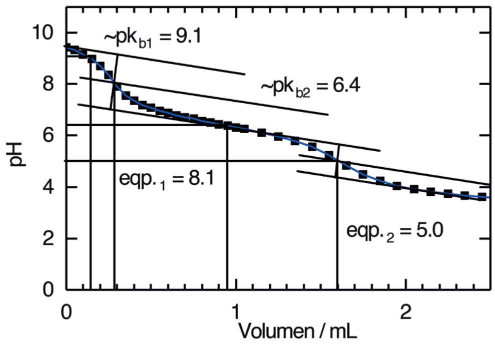

Abbildung S8. HCl (0,1 M) Titration einer Disperion von 26 mg PTI-LiBr (550 °C, 48 h). Der zweite Äquivalenzpunkt bei pH 5 entspricht dem pH-Bereich, in dem auch Veränderungen in den optischen Spektren stattfinden (Auftreten von 330 nm, 420 nm Photolumineszenz und 260 nm Absorptionsbande).

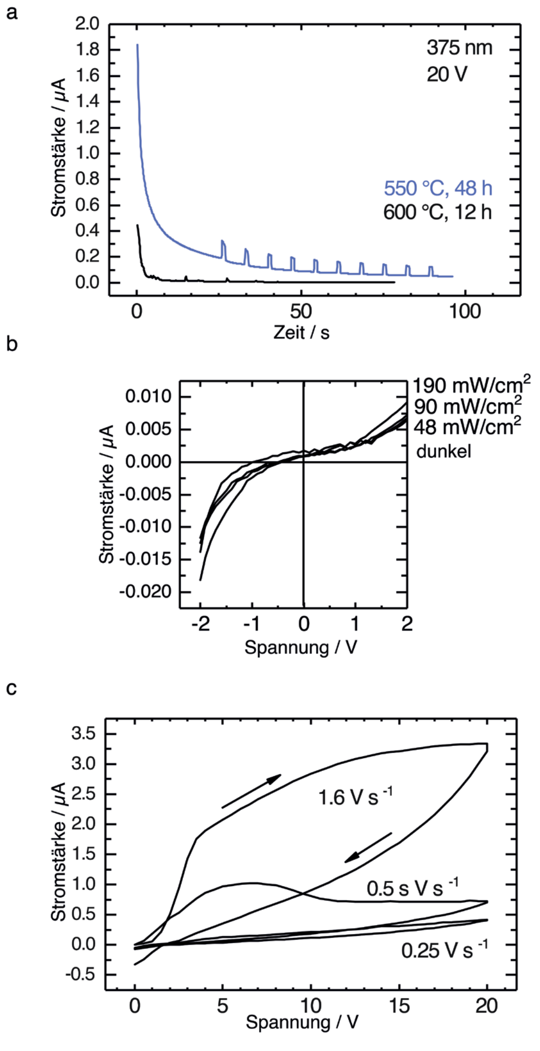

Abbildung S9. a) Vergleich des Fotoleiters mit PTI-LiBr bei 600 °C und 550 °C. Ladungstransport und Fotostrom sind im 600 °C-Material aufgrund der beobachteten teilweisen Karbonisierung (schwarze Linie) nicht vorhanden. In der Vorrichtung, die PTI-LiBr von 550 °C verwendet, kann der Strom den Kanal des interdigitalen Substrats durchqueren, und es wird ein Fotostrom beobachtet, wenn er mit einer 375-nm-LED bei 375 nm und einer Bestrahlungsstärke von 46 mW cm^{-2} angeregt wird. b) Bestrahlungsstärkeabhängiger

IV-Sweep der Fotoleitervorrichtung. Erhöhte Bestrahlungsstärke mit einer 375-nm-LED führt zu einem erhöhten Stromfluss. Der niedrige Strom ist das Ergebnis der polykristallinen Materialeigenschaften (Korngrenzen) und wahrscheinlicher energetischer Unordnung in teilweise interkalationsfreien Schichten. b) IV-Sweeps eines interdigitalen Bauelements unter Verwendung eines 550 °C, 48 h-Produkts mit unterschiedlicher Sweep-Geschwindigkeit. Die beobachtete Hysterese ist auf die Migration von Li+ und Br-Ionen zurückzuführen.

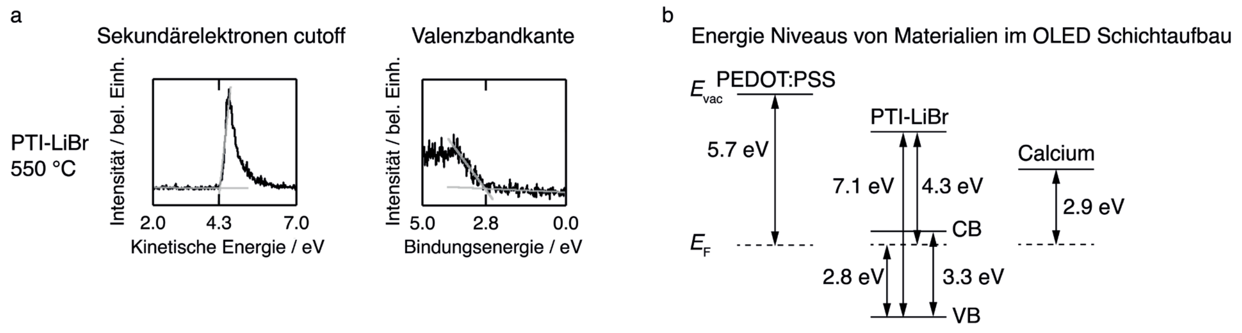

Abbildung S10. a) Ultraviolett-Photoelektronenspektroskopie (UPS) von PTI-LiBr auf ITO-Substraten. b) Energieniveaus von OLED-Materialien. Die Austrittsarbeit von PEDOT:PSS und Calcium wurden der Literatur entnommen (doi: 10.1063/1.2435350, doi: 10.1063/1.32353). Das Valenzband von PTI-LiBr wurde mittels UPS gemessen, während die Position des Leitungsbandes durch Kombination mit den UV-Vis-Spektroskopiedaten geschätzt wurde.
